# Women’s and practitioners’ views of early intensive decongestive lymphoedema treatment for breast cancer-related arm lymphoedema: a focus group study

**DOI:** 10.1007/s11764-025-01897-z

**Published:** 2025-12-02

**Authors:** Eunice Jeffs, Emma Ream, Yan-Shing Chang, Cath Taylor, Debra Bick

**Affiliations:** 1 Florence Nightingale Faculty of Nursing, Midwifery & Palliative Care, King’s College London, London, UK; 2 Dermatology Department, Guy’s & St Thomas’ NHS Foundation Trust, London, UK; 3 School of Health Sciences, University of Surrey, Guildford, UK; 4 Warwick Clinical Trials Unit, Warwick Medical School, University of Warwick, Coventry, UK

**Keywords:** Focus group, Breast cancer, Lymphoedema/lymphedema, Decongestive lymphoedema treatment, Outcomes

## Abstract

**Purpose:**

To explore the experiences and opinions of women with breast cancer-related arm lymphoedema (BCRL), a common sequela of breast cancer treatment, and of lymphoedema practitioners regarding current lymphoedema treatment in the UK, priority outcomes and assessment methods, and acceptability of a future trial of lymphoedema treatment and use of intensive-decongestive lymphoedema treatment (DLT) for women presenting with BCRL.

**Method:**

Two focus groups were conducted with 13 women and three focus groups with 13 lymphoedema practitioners. Discussions were transcribed and analysed using framework analysis.

**Results:**

Usual UK lymphoedema care was self-treatment with hosiery. Women and practitioners supported the notion of early intervention although practitioners reported lack of capacity to provide intensive-DLT. All groups considered arm volume an important outcome but were unsure what treatment outcomes could be expected. Participants supported the need for a trial to identify effective treatment for women with previously untreated BCRL but could not describe criteria for a good clinical outcome.

**Conclusions:**

Study findings demonstrate the need and desire of women and practitioners for robust research to determine the most effective treatment for women with early-BCRL, that is, within 12 months of BCRL onset, including intensive-DLT. However, UK lymphoedema services lack capacity to provide intensive-DLT, so the potential to conduct a future trial of effectiveness is likely to be limited.

**Implications for Cancer Survivors:**

Women with arm BCRL can expect to receive self-treatment with hosiery. There is a need to identify effective treatment for women with arm lymphoedema and criteria for a good clinical outcome.

**Supplementary Information:**

The online version contains supplementary material available at https://doi.org/10.1007/s11764-025-01897-z.

## Background

Breast cancer-related arm lymphoedema (BCRL) affects around 14% of women following breast cancer treatment [1]. Women experience a range of problems, including increased arm size, arm heaviness, difficulty with daily activities, psychological morbidity, and increased risk of infection [2–4]. BCRL is a problem regardless of the amount of visible swelling and whether symptoms are mild, moderate, or severe [5]. Women require treatment to manage BCRL symptoms which may over time become more challenging and difficult to treat [6, 7].

Much has been written about assessment and treatment of BCRL and measurement of treatment outcomes [8, 9], although systematic reviews of lymphoedema treatment effectiveness consistently conclude there is need for further research due to small sample sizes and heterogeneity of study populations, interventions, and outcomes [10]. Practitioners and researchers currently use a variety of assessment and treatment methods [11, 12], although the treatment outcomes that women with BCRL and lymphoedema practitioners consider important and the best way to measure these are uncertain [13–15].

Little is known about usual lymphoedema care women receive, their views on treatment offered or access to this. Few United Kingdom (UK) services have reported actual treatment provision [16–18]. International consensus recommends two-phase decongestive lymphoedema treatment (DLT) with initial intensive-DLT comprising several weeks of daily multi-layer compression bandaging (MLCB), manual lymph drainage (MLD), exercise, and skin care [19, 20]. However, UK best practice guidelines recommend self-treatment with compression hosiery for early/mild lymphoedema, reserving practitioner-delivered intensive-DLT to treat moderate-severe lymphoedema [21, 22].

A systematic review of evidence (from inception to July 2016) for treatment of BCRL within 12 months of symptom onset (early-BCRL) provided no evidence to justify change in lymphoedema treatment, whether self-treatment with hosiery (UK) or two-phase DLT typically offered by other countries [23]; findings were confirmed in a later review by the same research team [24]. Most studies are underpowered with wide variation in sample size, population characteristics, and assessment and treatment protocols. A relatively large trial of women with BCRL (*n* = 103) found no difference in outcomes for 2-phase DLT compared to self-treatment with hosiery alone, although their study population included women with any BCRL duration and an unknown number with previous experience of lymphoedema treatment [25]. Little is known about the experience of delivering or receiving intensive-DLT or barriers and facilitators to participating in lymphoedema research.

This paper reports a qualitative study which attempted to address some of these gaps in knowledge. The aim was to explore the perspectives of women with BCRL and lymphoedema practitioners (hereafter referred to as ‘women’ and ‘practitioners’) regarding UK treatment provision for women with any BCRL duration and participation in future lymphoedema research. Study objectives addressed key areas of uncertainty to identify: (1) ‘usual’ BCRL care in the UK; (2) value and acceptability of lymphoedema treatments; (3) priority outcomes; (4) meaningful methods of assessing treatment benefit; (5) acceptability of participating in a future trial; (6) potential burden of intensive-DLT. This study was conducted in preparation for developing a feasibility study of a definitive trial of BCRL treatment effectiveness (to be reported elsewhere).

## Method

Focus group discussion was undertaken to separately explore women’s and practitioners’ views and experiences of receiving and providing lymphoedema treatment in UK National Health Service (NHS) clinics. Supplementary Information 1 contains a completed Consolidated criteria for Reporting Qualitative research (COREQ) checklist [26].

### Participant recruitment

Women and practitioners were purposively selected for key characteristics and experiences, including lymphoedema duration and treatment provision [27]; see sampling matrix, Table 1. It was considered likely that a minimum of two groups for women and two groups for practitioners, with 6–8 participants per group, would generate sufficient data to meet the study objectives [28].

**Table 1:** Sampling matrix for women with BCRL and lymphoedema practitioners

Sampling criteria	Quota sought	Invited	
***Sample for women with BCRL***	12–20	101	
Lymphoedema treatment received			
Intensive-DLT Self-treatment	5 + 12 +	13 101	
Age at initial lymphoedema treatment			
20–39 40–59 60–74 75 +	1–5 5 + 5 + 1–5	4 47 41 9	
Ethnicity			
White Asian/Asian British Black/black British Other ethnic groups	5 + 1–5 1–8	65 7 21 8	
Work status at initial lymphoedema treatment			
Paid work Not working Student Retiree	5 + 1 1–3 5 +	*Unknown*	
Age of dependent children at initial lymphoedema treatment			
Pre-school Primary school Senior school None	1–5 1–5 1–5 5 +	*Unknown*	
***Sample for lymphoedema practitioners***	12–20	50	
Lymphoedema treatment provided			
Intensive-DLT Self-treatment	6 + 12 +	> 20 50	
Lymphoedema experience			
< 2 years^1^ 2–5 years^1^ > 5 years	3 + 3 + 3 +	> 15	
Type of lymphoedema service^2^			
Hospital-based Hospice-based Community-based	3 + 3 + 1 +	20 18 9	
Professional background^3^			
Nurse Physiotherapist Occupational therapist	5 + 3 + 1 +	30 6 0	

^1^Unknown at invitation whether < 2 years or 2–5 years. ^2^Unknown at invitation, *n* = 3. ^3^Unknown at invitation, *n = 14*

All records at one hospital-based lymphoedema clinic in central London (*n* = 403) were screened in early July 2015 (EJ). Women were ineligible if they had bilateral BCRL, had received cancer treatment within the previous three months, were receiving privately funded lymphoedema treatment, were unable to independently communicate in English, or had documented poor physical or mental health (see Supplementary Information 2). The researcher organised the 212 eligible women alphabetically by surname and sent a personalised letter and study information to the first 101 of 212 eligible women; age, gender, duration, and extent of BCRL are independent of surname, so there was no lymphoedema-related or personal bias affecting the selection. In addition, three relevant charities published study adverts: Breast Cancer Care, Lymphoedema Support Network, and Breast Cancer Haven. The minimum sample target was achieved with initial invitations and social media so, due to time constraints, the other 111 eligible women were not contacted and no reminders were sent.

Fifty lymphoedema practitioners from 33 lymphoedema services in London and the Home Counties were identified (EJ) and sent study information. The London lymphoedema Community of Practice and the British Lymphology Society (BLS) London regional group forwarded study information to all practitioners on their email circulation lists. Practitioners were ineligible if they had less than 6 months’ experience treating BCRL or worked *only* in a service which charged for BCRL treatment (see Supplementary Information 2).

**Fig. 1:**
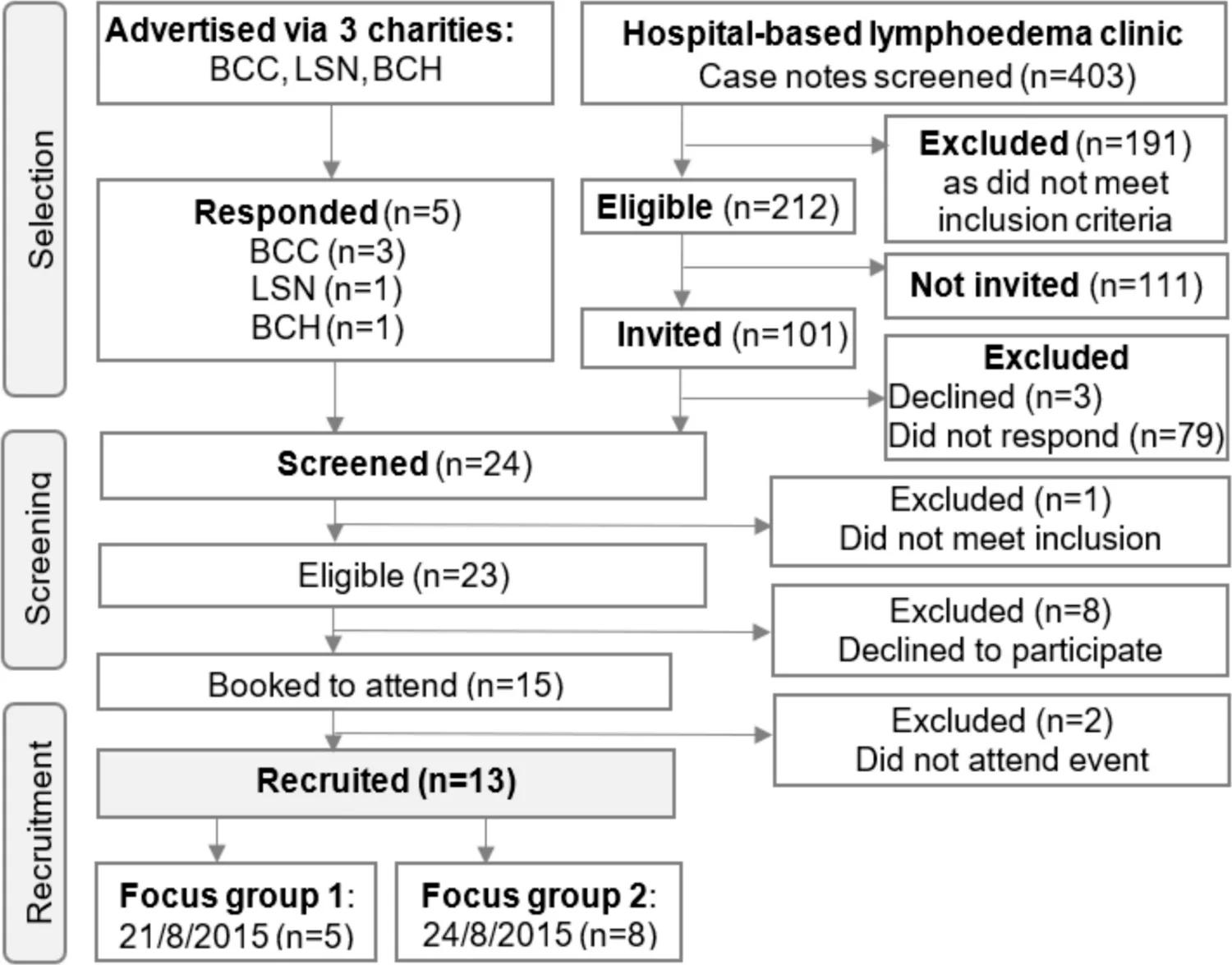

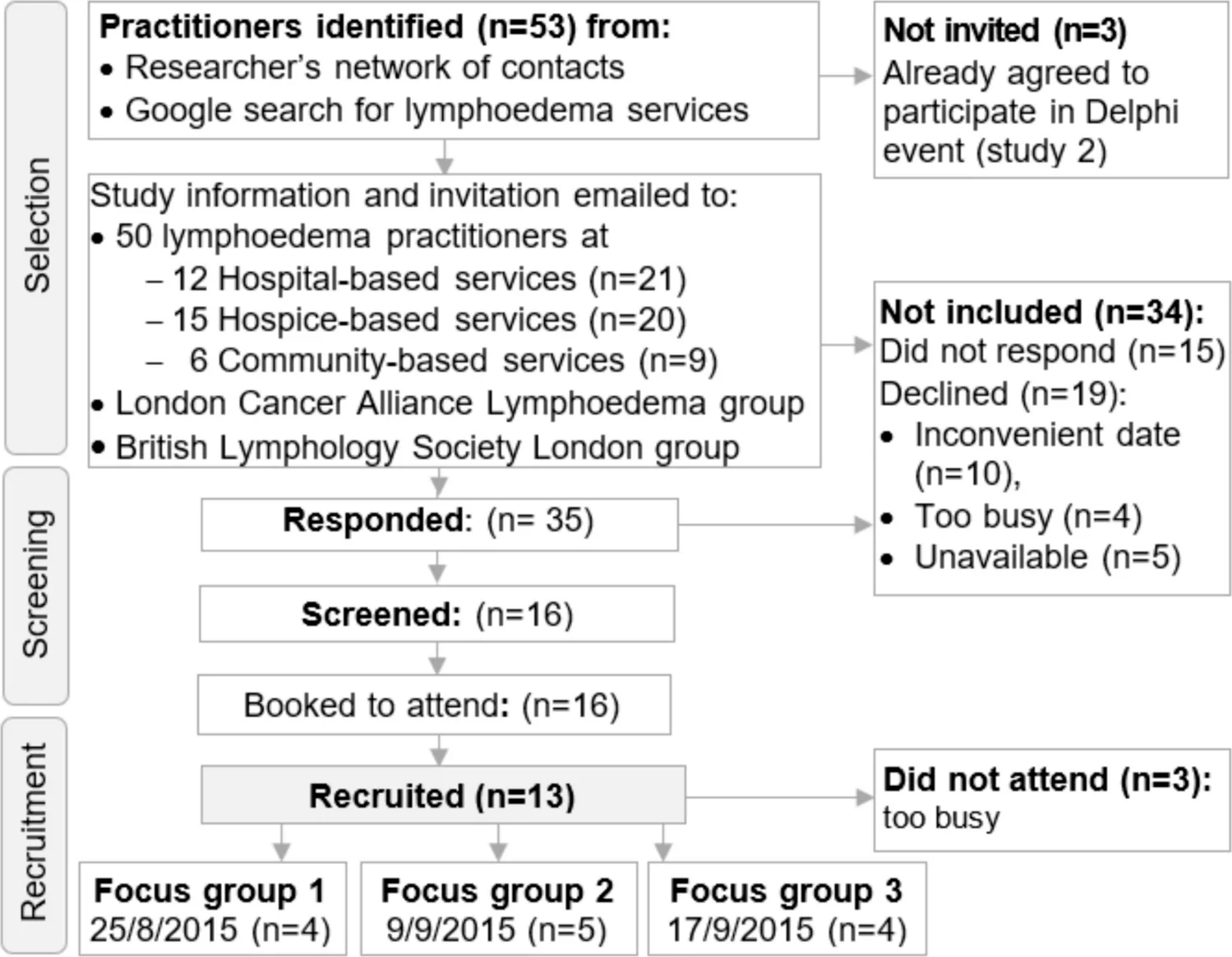
**a** Selection and recruitment of women. **b** Selection and recruitment of practitioners

Twenty-seven women and 35 practitioners responded (Fig. 1a and b); only one woman declined, with no reason provided. Eligibility was confirmed (EJ); one woman with breast oedema and no arm symptoms was excluded. Fifteen women and 16 practitioners were available to participate and allocated to focus groups by their preferred date and to ensure representation of relevant characteristics (Table 1) in each group.

### Data collection

Two topic guides to meet the study aims, with open questions, were developed (EJ) from reviewing available evidence, personal knowledge, and experience of lymphoedema treatment (Supplementary Information 3). Face validity was tested through discussion with the Lymphoedema Patient Reference Group. Consequently, pictures of treatment components were included for women unfamiliar with intensive-DLT.

Focus groups were held at a London-based university or hospital between 21st August and 17th September 2015. Participants attended only one group. Mean duration of focus group discussion was 83 min (range 79–86) for women and 94 min (range 81–107) for practitioners. Relevant topic guides were adapted (question order, topics) to group member experiences and flow of discussion. Discussions were audio-recorded with verbal permission of participants.

All discussion groups were facilitated by the researcher (EJ), a female lymphoedema expert and doctoral student with a pragmatic methodological orientation to research and clinical practice, training in focus group discussion, and no previous relationship with participants. The notetaker (YSC), an experienced qualitative female researcher with no prior knowledge of lymphoedema, mapped conversational flow. The researcher wrote field notes immediately following each discussion group. As doctoral project time constraints did not allow sufficient time to analyse data between sessions, the researcher also reflected with the notetaker on each focus group to review the content and flow of discussion and decide whether to amend the topic guides; only minor adjustments were required to improve wording and order of questions. Transcripts were professionally transcribed, verbatim but omitting pause fillers (um, er), and checked (EJ) against recordings and field notes to ensure correct attribution of conversation and spelling of lymphoedema terms.

### Analysis

Transcripts were manually coded, labelled, and analysed using framework analysis to manage and interpret data [29, 30]; the researcher initially used Nvivo software but found this stultifying, so the analysis was completed with manual coding using pen and paper. Through familiarisation with the data, the primary analyst (EJ) identified and labelled themes, grouped and sorted data to identify connections between themes and groups of subthemes and their relationship to the study objectives. From this, an initial coding framework was created for women and for practitioners (Supplementary Information 4) with findings summarised into matrices for each group (Supplementary Information 5) [29]. Analysis and interpretation focused on transparency, authenticity, and trustworthiness rather than generalisation or replication of findings, with group-level analysis to maintain context [31]. The coding framework and matrices were refined through reflection and discussion with note-taker and project supervisors (DB,CT,ER).

The second analyst (YSC) verified matrix summaries and their interpretation against a sample of transcripts and field notes, with minor differences in opinion resolved by discussion. The findings were also sense-checked with the Lymphoedema Patient Reference Group and a self-selecting group of participants; rather than asking participants to check their transcript, to minimise study burden all were invited to a participant validation event, November 2016, to sense-check and explore the authenticity and credibility of the preliminary findings and researcher’s interpretation and conclusions.

### Ethical approval

The study was reviewed and approved by the National Research Service London— London Bridge ethics committee (IRAS 161732). Written informed consent was obtained immediately prior to each focus group with additional verbal consent for audio-recording. Participants were asked to maintain confidentiality regarding personal or sensitive issues discussed. No incentives were offered, although travel was reimbursed and a £20 contribution towards childcare costs was offered.

## Findings

The two women’s groups (FGW) included 12 women recruited from a London-based lymphoedema service and one via social media advertisement. They reported minimal to moderate BCRL severity; three reported no visible swelling that day. The 13 practitioners in three groups (FGP), all female, represented nine lymphoedema services providing a comprehensive range of treatments. Physiotherapists were available for only one of the three events, although this was not expected to greatly impact findings as UK lymphoedema training is open to nurses and physiotherapists alike. Tables 2 and 3 show summary participant characteristics. The final thematic framework (Table 4) consisted of four main themes: (1) ‘usual’ care received by/offered to women with arm BCRL, (2) monitoring treatment outcomes, (3) acceptability of proposed study, and (4) living with/managing lymphoedema. Individual and group findings for women and practitioners are summarised in matrices (Supplementary Information 5).

**Table 2:** Characteristics of women’s focus group participants

Characteristics		Group 1 (*n* = 5)	Group 2 (*n* = 8)
Age group	• 40–59 years • 60–75 years	2 3	2 6
Swelling	• Unilateral arm • Bilateral arm • Co-existing breast oedema	4 1 1	8 0 1
BCRL duration	*Median* years (range)	2.0 (2.0–10.0)	5.5 (0.5–12.0)
Perceived severity	• No swelling that day • Mild • Moderate • Severe	1 1 3 0	2 4 2 0
Perceived impact	• Not bothered by it • Bothered a little • Moderately bothered • Bothered a lot	1 2 1 1	1 1 6 0
BCRL treatment received	• Self-care only • Self-care + DLT^1^ ± IPCT^1^ • Self-care + IPCT^1^ + MLD^1^ • Self-care + MLD^1^	4 1 0 0	5 1 1 1
Ethnicity	• White British • Black British	4 1	8 0
Employment	• Part-time/full-time work • Retired/not working	1 4	3 5
Responsibility for dependent children at BCRL referral		1	1^2^

^1^provided by practitioner; ^2^regular carer for grandchildren

**Table 3:** Characteristics of practitioners’ focus group participants

Characteristics		Group 1 (*n* = 4)	Group 2 (*n = *5)	Group 3 (*n = *4)
Experience treating BCRL	• 2–5 years • > 5 years	2 2	1 4	0 4
Groups treated by lymphoedema service	• Cancer only • Cancer, non-cancer	3 1	2 3	2 2
Profession	• Nurse • Physiotherapist • Occupational therapist	4 0 0	1 4 0	4 0 0
Type of lymphoedema service	• Any Qualified Provider • Community-based • Hospice-based • Hospice + community-based • Hospital-based	0 0 0 1 3	1 0 1 0 3	0 1 0 1 2

**Table 4:** Final thematic framework for women and practitioners

Women’s themes		Practitioners’ themes	
***1.0***	***‘Usual’ care for women with arm BCRL***	***1.0***	***‘Usual’ care offered to women with arm BCRL***
1.1	Current lymphoedema treatment	1.1	‘Usual’ lymphoedema treatment
1.2	Variations to lymphoedema treatment	1.2	Variations to ‘usual’ BCRL treatment
1.3	What is treatment?	1.3	Women’s adherence to recommended treatment
1.4	Adherence to recommended lymphoedema treatment	1.4	When is intensive-DLT offered?
1.5	Burden of self-treatment, ‘usual’ care	1.5	What does the term ‘treatment’ mean?
***2.0***	***Monitoring treatment outcomes***	***2.0***	***Monitoring treatment outcomes***
2.1	Assessment of lymphoedema	2.1	Assessment methods used
2.2	Challenges for monitoring	2.2	Challenges for assessment and monitoring
2.3	Perceived lymphoedema treatment outcomes	2.3	Expectations of treatment 2.3.1. Self-treatment 2.3.2. Intensive-DLT
2.4	Priority outcomes	2.4	Priority outcomes
***3.0***	***Acceptability of proposed study***	***3.0***	***Acceptability of proposed study***
3.1	Acceptability of participation in future lymphoedema research	3.1	Acceptability of proposed lymphoedema trial
3.2	Burden of proposed study interventions	3.2	Burden of proposed study interventions
3.3	Treating early-BCRL with intensive-DLT – is it worth it?	3.3	Potential benefit of initial intensive-DLT
***4.0***	***Living with lymphoedema***	***4.0***	***Managing lymphoedema***
4.1	Physical and psychological consequences of BCRL	4.1	Characteristics of women newly presenting for BCRL treatment
4.2	Learning to live with lymphoedema	4.2	Changing nature of lymphoedema practice
4.3	Hopes, expectations, and beliefs about lymphoedema treatment	4.3	Challenges and frustrations of managing limited resources
4.4	Impact of knowledge deficit	4.4	Patient education

### ‘Usual’ BCRL care in the UK

All groups agreed that NHS usual BCRL care was self-treatment, with compression hosiery, education to support effective self-management, and monitoring by practitioners. All women-participants wore compression armsleeves although few mentioned skincare, exercise or SLD; practitioners offered SLD only where clinical need was combined with perceived receptivity to learning the technique, although women taught SLD were uncertain how to do it. Usual care did not include practitioner-delivered treatments such as intensive-DLT or MLD; only four women had ever experienced any form of intensive practitioner-delivered treatment.

Selection of treatment components was influenced by practitioner and patient preference, budget constraints, and availability of skilled personnel. Practitioners typically offered women with mild oedema a lightweight, class one, round-knit compression armsleeve, whereas women with larger limbs, distorted arm shape, firm or fibrosed oedema were given stronger, stiffer flat-knit garments. Practitioners adjusted hosiery to maximise improvement and control of BCRL symptoms. Women with poorly controlled symptoms or struggling with self-management were offered more frequent reviews subject to staff availability. Women were discharged once stable, with hosiery provision via GP prescription. Few practitioners mentioned evidence to support their clinical decision-making, and none cited specific research findings.

### Value and acceptability of lymphoedema treatments

Women showed varying awareness of options for managing BCRL, whether self-treatment or practitioner-delivered treatments. Some in FGW2 suggested self-treatment was not ‘treatment’ as the potential to improve swelling was limited, whereas practitioner-delivered interventions, such as MLCB and MLD, achieved an improvement so were treatment; the four women given intensive-DLT reported it was beneficial although might have been more effective if applied earlier.

W12 “I think when I’ve had the bigger interventions, as it were, I felt at least I’m doing something. Whereas just the sleeve thing doesn’t feel sometimes quite enough. You want more.” ((Agreement)) [FGW2]

Although all practitioner-groups agreed intensive-DLT should be offered as early as possible, to minimise progression of lymphoedema, none had sufficient resources to do so. Only a few believed women should be offered intensive-DLT as initial treatment.

“I do believe the important thing is early treatment, because if you just put them in the sleeve, and then you can’t see them for another few months, they could have developed fibrosis by then or deep thickening of the tissues. And then it becomes a battle because you’ve got no spaces and can’t book them in for intensive treatment.” [PP4, FGP2]

Practitioners reported increased demands on their services which had been largely managed using self-treatment with hosiery and by delaying or not offering intensive-DLT. As women were now referred earlier for treatment than in previous years, typically within a few months of developing symptoms, with minimal and more easily treated BCRL, self-treatment with hosiery was viewed as acceptable treatment. Hosiery prescription was adjusted to achieve a reduction in swelling.

All groups acknowledged challenges with adherence to recommended treatment, particularly hosiery. Women considered themselves largely adherent to self-treatment, wearing hosiery most days and exercising regularly. They did not view ‘time off’ wearing hosiery for special occasions as non-adherence and seemed unaware of the potential consequences for long-term control of BCRL.

Improved access to prescription hosiery enabled practitioners to discharge women with stable swelling and transfer long-term hosiery provision to community providers. However, all groups reported frequent delays and incorrect garments from local pharmacies, likely due to community healthcare staff lacking necessary knowledge. Practitioners were frustrated by the considerable clinic time required to deal with resultant problems.

W1 “Each time I asked for a prescription, my GP said they cannot find it, they can’t prescribe it. (…) when I took it to the pharmacist, I stood there for like an hour, she was on her computer (and) could not get the right prescription.”

W3“Yeah. I had the same thing.” [FGW1]

### Priority outcomes for study and methods of assessing treatment benefit

Women and practitioners identified arm volume as the priority outcome for a future study, although they were unable to quantify a clinically meaningful change. Practitioners could not identify another key outcome for research, whereas women included arm appearance and BCRL impact on daily activities and functional ability. In addition, women agreed that four issues identified by women in a previous study [32] could inform research outcomes and improve adherence to self-treatment by increasing patient understanding of the significance of BCRL changes through self-monitoring size, appearance, limb texture, and internal sensations.

“They sound so sensible. (…) I’m sure if I’d done that regularly I would be a lot more informed about lymphedema and how I should treat it.” [W8, FGW2]

Women also suggested practitioners should monitor lymphoedema treatment burden to determine its importance for monitoring in future research.

Practitioners were frustrated by the lack of objective tools to measure pitting oedema and fibrosis and difficulty measuring minimal lymphoedema. Few measured interstitial fluid or used tonometry. While some used health-related quality of life (HRQOL) questionnaires, women reported these did not distinguish between the impact on daily life of BCRL and its treatment and of avoiding activities which could exacerbate symptoms.

“Whenever you go to the clinic there is this survey about how you feel and you rate but there is nowhere for you to explain.” [W1, FGW1]

Practitioners could not describe optimal treatment outcomes but agreed delaying provision of intensive–DLT would not achieve an optimal outcome.

“I think that if patients with confirmed lymphedema were offered DLT at the first contact with the clinic it would have a very beneficial effect, because we do see the changes as time goes by… creating the cementing effect within the tissue and this cannot be reversed. … It leads into the hard type of tissue and … there’s no way to drain the fluid through that... So the sooner the better I think.” [PN1, FGP1]

### Acceptability of proposed study and potential burden of intensive-DLT

Women and practitioners agreed the proposed trial, comparing intensive-DLT with usual care for women with early-BCRL, was needed but expressed ambivalence about the acceptability of the intervention. Practitioners questioned whether women with early-BCRL are practically and psychologically ready for the burden of daily intensive-DLT, although the women considered daily travel for treatment a minimal burden. All groups acknowledged the challenge of wearing bulky bandages, but there were different opinions amongst the women regarding the burden and acceptability of intensive-DLT as initial treatment or study intervention.

W12 “It would be an interesting one to know if I had had (intensive-DLT) as standard treatment at the very beginning of the first diagnosis whether I would have developed it more seriously later.” (…)

W11 “To be honest I think the bandaging looks positively medieval and not 21^st^ century medical science. So, the thought of having to do that if you aren’t in enough discomfort really puts you off. If it was offered to me, no.”

W13 “To be honest with you, I would certainly have (had intensive-DLT) if I wasn’t able to type anymore, because that is so fundamental to my working life. But with my symptoms as they are, no, unless two weeks of it would guarantee that I wouldn’t have any more symptoms ever again - then that would be a small price to pay.” [FGW2]

Women suggested daily intermittent pneumatic compression therapy (IPCT), a ‘pump’, with hosiery worn between treatments, would have minimal impact on daily activities so was a more acceptable intervention than MLCB; of note, one woman (FGW2) suggested this combination before the researcher had presented it.

W12 “I wonder whether the pump one would be an easier treatment to accept at that point, perhaps a daily pump, because it’s less intrusive. (…)”

W11 “Because it’s more akin to radiotherapy: you’re just travelling in every day. You’ve done it; you’ve already been there.” (…)

W6 “A pump you can just walk away from after your time is up, can’t you?... But the bandage you’ve got on all the time.” [FGW2]

Practitioners had little or no experience of using IPCT but also viewed modified intensive-DLT, without MLCB, as more acceptable.

Women suggested willingness to participate in a future trial could be influenced by perceived lymphoedema severity, personal treatment preferences, and the requirement to wear long-term hosiery post-intervention even if allocated to intensive-DLT. However, their discussion revealed mixed understanding of the purpose of research and randomisation.

W1 “I’m wondering, how will a computer know the right treatment to decide for me?”

W5 “It doesn’t, it’s a trial. (…) If it was at the very beginning and you didn’t know anything about what was likely to be the result of wearing sleeves or any of the treatments, I think you would probably accept that, yes you would say, “Yes I’m happy” because you would believe then that anything that the clinicians or nurses or whoever said is likely to work.” [FGW1]

Practitioners reported that, although willing to participate in future lymphoedema research, services lacked capacity to deliver the study intervention even with reimbursement of additional treatment costs [33]. Furthermore, they expressed concern that implementation of future study findings could be difficult because practitioners would question the ethics of offering initial intensive-DLT to women with early-BCRL when patients with severe lymphoedema were waiting for intensive-DLT.

“You might find clinicians who feel they’re not able to provide the intensive treatment to those they want to, so to then be offering it to those (with early-BCRL) that would not meet their criteria!” [PP1, FGP2]

### Living with lymphoedema

Women were not specifically asked about living with lymphoedema but their experiences were woven throughout the discussions. All women reported fluctuating symptom severity, changes to their swollen arm appearance, with BCRL impacting their daily activities, arm function and employment. They talked at length about challenges and frustrations associated with treatment, particularly accessing follow-up appointments, wearing and obtaining hosiery which impacted daily life and routine activities and affected fit of clothes, and how hosiery was difficult to keep clean and dry when preparing food and toileting.

The psychological impact of lymphoedema was considerable, with BCRL a daily reminder of breast cancer and some women reporting significantly altered body image. Women believed hosiery revealed their lymphoedema and invited public questions about something personal and private.

W1 “It’s so awful most times, like when I walk in at school time, the kids, “Oh Miss, why have you got this on?” The questions, all the time...”

W4 “You have hit a point with me in that I’m sure we’re all in the same boat where you’re always getting people in shops or wherever ((W2 and W3: ‘Yeah’)) saying, ‘Oh what have you done to your hand’” [FGW1]

While many appeared to have accepted the life-long nature of BCRL and, regardless of symptom severity, had assimilated self-treatment into daily life, others had not. For example, some women used activities as daily exercise while wearing hosiery to improve symptom control and condition their arms, whereas many limited their activities because they were unsure how much they could safely do.

W5 “I manage to do most things. Ironing I don’t particularly like, but I do it because I believe any form of housework helps, as long as it’s not really excessive.” (…)

W4 “We’re aware of what might happen and therefore we let other people do these things. We are naturally hesitant about doing the ironing or whatever.” [FGW1]

Women wanted to better understand how to recognise and interpret swelling changes and consequently adjust their self-treatment. They received considerable information from practitioners and through internet searches but were frustrated by their limited knowledge of treatment options and what to expect from treatment. Some questioned whether practitioners knew what outcomes were achievable and thought they did not offer much hope of improvement.

“I’m feeling quite annoyed actually that there is such a bleak prognosis given – that it’s only going to get worse and worse and that’s it. And actually I would rather (be told): ‘the truth is we don’t really know’. ((Laughs)) I would find that (a) more hopeful, and (b) more truthful.” [W13, FGW2]

### Participant validation event

Seven women and four practitioners attended the participant validation event, including women with early-BCRL and at least one representative from each group (see Supplementary Information 6). Participants confirmed the key preliminary findings (Supplementary Information 7) reflected their group discussion and personal experiences. Subsequent discussion explored four issues they thought could improve clinical outcomes with implications for the success of a future definitive trial: (1) identifying reasonable lymphoedema treatment expectations; (2) the value of good communication and education to help women make informed decisions about adherence to self-treatment, particularly hosiery, and understand the study purpose; (3) the burden of wearing hosiery and potential impact on fidelity to a future trial protocol; and (4) the potential impact of GP hosiery prescription, with delays and inaccuracies, on delivering the trial intervention.

A new finding was that women reported boosted morale from hearing other participants’ descriptions of BCRL self-management. They suggested a buddy system, pairing new and experienced patients, would increase understanding of lymphoedema and its treatment, thereby improving clinical outcomes and trial retention.

“Along the way you can find out by trial and error just exactly what you can do (but) it’s almost as if each patient needs an individual sort of friend, pal, to take them along.” [W4, >10-year BCRL duration]

## Discussion

These study findings provide new knowledge about women’s and practitioners’ views on BCRL treatment in the UK and their expected and perceived outcomes of treatment which could inform clinical practice and development of future research protocols; although the study was conducted in 2015, these findings are still relevant as there have been no substantial changes to UK lymphoedema management over the past 10 years. These findings add to existing knowledge about characteristics of BCRL, the experience of living with BCRL and burden of treatment [34–36]. This is the first study to report the actual treatment practitioners offer and women receive from UK lymphoedema clinics. The finding that the UK treatment approach is to offer all patients self-treatment with hosiery as usual care is contrary to international guidelines recommending two-phase DLT [19]. Earlier reports from UK treatment services document that intensive DLT was provided only to those with more severe symptoms [17, 37]. Our findings clearly show that UK services lack capacity to provide two-phase DLT as part of standard care, regardless of lymphoedema severity, with patients instead receiving minimum care, that is, instruction for self-treatment with hosiery. Schulze et al. [38] suggested the UK may be unique in offering self-treatment as standard care. Publications from the LIMPRINT project revealed that the UK has the highest use of hosiery (92%) and lowest provision of MLCB (8%) compared with 39–76% hosiery and 30–85% MLCB provision in five other countries: Canada, Denmark, Italy, Japan, and Turkey [39–44]. While more recent papers describe alternatives to UK practitioner-delivered treatment, such as adjustable compression wrap devices (ACWD), these are also self-treatment components [45–47].

Participant concerns regarding the timely provision of correct compression garments have been reported by other UK practitioners [48, 49]. Delays to hosiery provision during a clinical trial would affect outcomes, although the degree of impact is unknown. Brown and Sneddon [50] surveyed UK practitioners (*n = *111) and found 50% had experienced problems with hosiery provision causing moderate-severe impact on patient care and outcomes. A procurement model, with lymphoedema clinics purchasing and providing garments, has been presented by two different UK lymphoedema services as a solution with economic benefits as well as improving the patient experience [48, 51] although this will require considerable organisational change. In addition, recent expansion of UK patient prescription services reportedly reduces errors and delays in receiving garments and the associated risk of harm to patients [52]. Challenges with obtaining hosiery are not unique to the UK, although reasons may differ in countries which require women to pay all or some of the hosiery cost [42, 53, 54].

There is no consensus regarding standardising use of compression garments in research, with studies reporting a variety of methods; for example, Tambour et al. [68] provided custom-made garments without reporting compression strength, Dayes et al. [25] used garments providing 30–40 mmHg, whereas McNeely et al. [69] reported garments providing at least 30 mmHg. Our study found practitioners typically used a wide range of compression garments to achieve comparable benefit, with individual prescriptions adjusted (compression strength, material stiffness, manufacturer) according to clinical need. This suggests a future trial should utilise real-life hosiery prescriptions, according to the practitioner’s clinical judgement, with careful documentation of each garment to facilitate monitoring and interpretation of treatment outcomes.

An incidental new finding of this study was that women were largely unaware of the impact that varying their self-treatment behaviours had on BCRL symptom control and treatment outcomes. It is likely that the small, incremental changes associated with non-adherence to wearing hosiery may be overlooked because fluctuation in oedema symptoms is common [43]. Women in other studies have identified symptom changes that could be used to determine swelling improvement or deterioration, although the impact on treatment adherence has not been explored [32, 55].

Women and practitioners in this study valued monitoring of arm volume but were ambivalent about the value of self-report questionnaires which did not address personal issues of significance, such as impact of BCRL treatment. Although most lymphoedema studies report changes to arm volume, there is no consensus regarding clinically meaningful outcomes or minimal important differences for arm volume or other outcomes, and variation in tools and methods makes comparisons between studies difficult [13, 56]. Tools are lacking to monitor the burden of lymphoedema treatment and adherence to self-treatment; although some self-report questionnaires address both symptom severity and impact on function and HRQOL, such as the Lymphedema Symptom Intensity and Distress Scale-Arm [57], these are not designed to support self-treatment and self-management of progress.

An unexpected new finding was that practitioners and women were uncertain about treatment expectations, and women questioned whether practitioners lacked knowledge about expected outcomes or merely the skills to clearly articulate treatment goals. A recent international survey reported the top three successful treatment outcomes as stable limb volume, improved HRQOL, and controlled symptoms, with practitioners rating highly the patient’s ability to self-manage swelling with professional support [58]. In contrast, other studies reported a desire for reduced swelling and improved BCRL symptoms and defined successful intensive-DLT as improvement or disappearance of swelling [59, 60]. Some practitioners in this study expressed concern that underlying changes in subcutaneous tissues could not be adequately addressed by self-treatment with hosiery, thus allowing development of chronic symptoms including thickened subcutaneous tissues. Bundred et al. [6] found that, of 187 women surveilled over a 22-month period following breast cancer treatment, 16% progressed from mild to moderate BCRL despite being fitted with hosiery. The UK approach of initial treatment with hosiery may remove the fluid component but is unlikely to change the underlying fibrotic and adipose tissue, perpetuating the problem of fluctuating lymphoedema symptoms. Fatty tissues and fibrosis will reduce the effectiveness of compression [61–63].

There is a need for robust studies to address the effectiveness of BCRL treatment, particularly the effectiveness of intensive-DLT as initial treatment [24]. A Delphi survey of UK practitioners and patients found the highest research priority was to determine which early intervention modalities are most effective to prevent or control lymphedema and prevent long-term complications [64]. However, our study findings highlight some challenges to conducting treatment effectiveness studies in the UK, including a lack of capacity to provide practitioner-delivered study interventions of MLCB and MLD. Several reports highlight UK services struggling to meet demand for treatment, with increased referrals for complex non-cancer-related lymphoedema using substantial treatment resources and long wait lists for intensive-DLT [65–67]. Brown and Sneddon [50] reported that most lymphoedema services had fewer than five practitioners, which limits capacity for involvement with projects outside core service delivery.

Burden of intensive-DLT, particularly bulky and restrictive MLCB, and of visible hosiery has been reported elsewhere [70–72], with implications for both clinical practice and research. Our study findings suggest the burden of MLCB could affect recruitment to clinical trials whereas non-adherence to recommended hosiery is a risk to trial protocol fidelity for both comparator and intervention groups in the post-intervention period. Reported adherence to recommended treatment varies between studies and according to treatment component, so adherence to components of the study intervention and comparator should be carefully documented [43, 73, 74].

### Strengths and limitations

A key strength is the inclusion of participants with a range of experience and characteristics who were representative of women with BCRL and UK practitioners and remained engaged throughout the event. Another strength is the participant validation event addressing the authenticity of preliminary findings and researcher interpretation. Although data collection was conducted in 2015, there have been few changes to UK lymphoedema service provision so the findings are still relevant.

Limitations include lack of women under 40 and over 75 years, with under-representation of women of colour (global ethnic majority), those with early-BCRL, and those with experience of intensive-DLT, which could affect the generalisability of the findings to these groups; this largely reflects the UK breast cancer and BCRL populations although the impact on study findings is unknown. It is not known whether selecting eligible women in alphabetical order of surname introduced any bias as demographic and lymphoedema-related characteristics are independent factors; inviting all eligible women would have increased rigour. The under-representation of practitioners with less than 2 years’ lymphoedema experience is unlikely to impact on study findings as practitioners in each group had enough experience to discuss the topic questions. There was insufficient time to analyse data between sessions and confirm data saturation with additional discussion groups so, had time and resources allowed, a further discussion group for both women and practitioner groups could have explored whether there were issues to be further probed.

## Conclusion

These findings demonstrate the need and desire of women and practitioners for robust research to determine the most effective treatment for women with early-BCRL, and will be of interest to both clinicians and researchers. Participants agreed a future trial to determine the effectiveness of intensive-DLT for women newly presenting with BCRL was important, although they suggested reducing the trial burden with an alternative to MLCB, such as IPCT plus 24-hour hosiery. Key components of UK ‘usual’ care, the comparator in a future trial, were clearly identified as self-treatment, with the importance highlighted of carefully documenting all aspects of treatment to describe both intervention and comparator and monitor their impact on outcomes. While participants agreed the priority outcome for a future definitive trial was arm volume, markers of successful treatment were not determined, and women highlighted challenges for monitoring the impact of BCRL and its treatment on arm function and HRQOL.

Three important issues requiring further investigation before undertaking a definitive trial were highlighted by practitioners who questioned the appropriateness of initial intensive-DLT for women with mild symptoms: (1) expectations of treatment and perceived value of intensive-DLT; (2) lack of service capacity to deliver the intensive-DLT; (3) clinical implications of the UK’s reliance on self-treatment with hosiery.

## Supplementary Information

Below is the link to the electronic supplementary material.

ESM1 (PDF 24.7 KB)

ESM2 (PDF 28.3 KB)

ESM3 (PDF 494 KB)

ESM4 (PDF 33.5 KB)

ESM5 (PDF 349 KB)

ESM 6 (PDF 19.7 KB)

ESM 7 (PDF 22.2 KB)

## Data Availability

All data supporting the findings of this study are available within the paper or the women and practitioner matrices (within the Supplementary Information).
