# Supplementary material for: Women’s and practitioners’ views of early intensive decongestive lymphoedema treatment for breast cancer-related arm lymphoedema: a focus group study
Source: J Cancer Surviv. 2025 Dec 2;20(5):2460–73. doi: 10.1007/s11764-025-01897-z (PMC13723537; doi:10.1007/s11764-025-01897-z)
Supplement: Supplementary file 1 [file 11764_2025_1897_MOESM1_ESM.pdf]

Supplementary Information 1: Consolidated Criteria for Reporting Qualitative Studies (COREQ) checklist for focus group discussion

Section and Topic	Item #	Checklist item	Reported page on #
**Domain 1: research team and reflexivity**			
Personal characteristics			
Interviewer/facilitator	1	Which author/s conducted the interview of focus group?	7-8
Credentials	2	What were the researcher's credentials?	7-8
Occupation	3	What was their occupation at the time of the study?	7
Gender	4	Was the researcher male or female?	7
Experience and training	5	What research or training did the researcher have?	8
Relationship with participants			
Relationship established	6	Was a relationship established prior to study commencement?	8
Participant knowledge of the interviewer	7	What did the participants know about the researcher?	NR
Interviewer characteristics	8	What characteristics were reported about the interviewer/facilitator?	7-8
**Domain 2: study design**			
Theoretical framework			
Methodological orientation and theory	9	What methodological orientation was used to underpin the study?	7
Participant selection			
Sampling	10	How were participants selected?	4, Table 1
Method of approach	11	How were participants approached?	4
Sample size	12	How many participants were in the study?	5-7
Non-participation	13	How many people refused to participate or dropped out? Reasons?	5
Setting			
Setting of data collection	14	Where was the data collected?	7
Presence of non-participants	15	Was anyone else present besides the participants and researcher?	8

Description of sample	16	What are the important characteristics of the sample?	9-10, Table 3a,b
Data collection			
Interview guide	17	Were questions, prompts, guides provided by the authors? Was it pilot tested?	7, Suppl Info 3
Repeat interviews	18	Were repeat interviews carried out?	7
Audio/visual recording	19	Did the research use audio or visual recording to collect the data?	8
Field notes	20	Were field notes made during and/or after the interview or focus group?	8
Duration	21	What was the duration of the interviews or focus group?	7
Data saturation	22	Was data saturation discussed?	23
Transcripts returned	23	Were transcripts returned to participants for comment and/or correction?	8
**Domain 3: analysis and findings**			
Data analysis			
Number of data coders	24	How many data coders coded the data?	8
Description of the coding tree	25	Did authors provide a description of the coding tree?	Table 1, Suppl Info 4
Derivation of themes	26	Were themes identified in advance or derived from the data?	8
Software	27	What software, if applicable, was used to manage the data?	8
Participant checking	28	Did participants provide feedback on the findings?	8, 18-19
Reporting			
Quotations presented	29	Were participant quotations presented to illustrate the themes/findings? Was each quotation identified?	12-19
Data and findings consistent	30	Was there consistency between the data presented and the findings?	11-19. Suppl Info 5
Clarity of major themes	31	Were major themes clearly presented in the findings?	9-18
Clarity of minor themes	32	Is there a description of diverse cases or discussion of minor themes?	

Title: Women's and practitioners' views of early intensive decongestive lymphoedema treatment for breast cancer-related arm lymphoedema: a focus group study.

Journal of Cancer Survivorship.

Authors: Jeffs E, Ream E, Chang YS, Taylor C, Bick D. corresponding author: Jeffs E. King's College London and Guy's & St Thomas' NHS Foundation Trust.
Eunice.jeffs@kcl.ac.uk; Tel: 07557325642
