# Supplementary material for: Women’s and practitioners’ views of early intensive decongestive lymphoedema treatment for breast cancer-related arm lymphoedema: a focus group study
Source: J Cancer Surviv. 2025 Dec 2;20(5):2460–73. doi: 10.1007/s11764-025-01897-z (PMC13723537; doi:10.1007/s11764-025-01897-z)
Supplement: Supplementary file 2 [file 11764_2025_1897_MOESM2_ESM.pdf]

Supplementary Information 2: Eligibility criteria for women with BCRL and lymphoedema practitioners

Inclusion	Exclusion	Rationale for exclusion
For women with BCRL		
BCRL, any duration or severity	Lymphoedema affecting only hand, chest wall, breast	No co-existing arm lymphoedema
Completed cancer treatment ≥3 months ago	Breast cancer treatment <3 months ago: surgery, radiotherapy, chemotherapy	May be emotionally vulnerable if recovering from treatment. Likely to focus on cancer-related issues
Aged ≥18 years	Children	Would be excluded in future study; breast cancer is rare in adolescents and young people (McVeigh et al, 2021)
	Majority BCRL treatment privately funded	Future research will address NHS treatment provision
	Bilateral BCRL	No unaffected arm for comparison
	Unable to independently communicate in English	Translation process would disrupt and limit group discussion. Authenticity could not be verified during event
	Documented psychological issues at screening, e.g. depression, dementia	Potential cognitive impairment may affect ability to discuss topic. Woman may focus on unrelated issues
	Documented poor health at screening, e.g. recent in-patient stay, multiple appointments for another health issue	Woman may focus on issues unrelated to lymphoedema
	Attended only one lymphoedema appointment	Limited experience of lymphoedema treatment
	Documented treatment non-adherence, e.g. no BCRL attendance within past 2yrs	Unknown impact of treatment non-adherence on BCRL experience
For lymphoedema practitioners		
Registered HCP or MLD therapist registered with MLDUK.org	Practitioner is not registered HCP or MLD therapist registered with MLDUK.org	Therapists who are not recognised lymphoedema practitioners may use different treatments
At least 6 months experience treating BCRL (or equivalent part-time experience)	Less than 6 months experience treating BCRL (or equivalent part-time experience)	Insufficient experience to meaningfully contribute to all relevant issues
Provider of BCRL treatment in non-fee-paying service, hospital, hospice, community-based	Work only in service which charges for BCRL treatment	Private practitioners have access to wider range of treatment than NHS-based practitioners

Title: Women's and practitioners' views of early intensive decongestive lymphoedema treatment for breast cancer-related arm lymphoedema: a focus group study.

Journal of Cancer Survivorship.
Authors: Jeffs E, Ream E, Chang YS, Taylor C, Bick D. corresponding author: Jeffs E. King's College London and Guy's & St Thomas' NHS Foundation Trust. Eunice.jeffs@kcl.ac.uk; Tel: 07557325642
