# Supplementary material for: Women’s and practitioners’ views of early intensive decongestive lymphoedema treatment for breast cancer-related arm lymphoedema: a focus group study
Source: J Cancer Surviv. 2025 Dec 2;20(5):2460–73. doi: 10.1007/s11764-025-01897-z (PMC13723537; doi:10.1007/s11764-025-01897-z)
Supplement: Supplementary file 3 [file 11764_2025_1897_MOESM3_ESM.pdf]

Supplementary Information 3: Exemplar focus group topic guides

(a) Exemplar focus group topic guide for women

(questions to be adapted according to group and, where appropriate, amended as the focus groups progress)

1. Introduction – of research team members and the format of the event, etc.

2. Background

Can you tell me:

- How long have you had lymphoedema?
- What lymphoedema treatment have you had? *[?show pictures of treatment as prompts?]*

3. Experiences of lymphoedema treatment

Thinking back to when you first developed lymphoedema, can you tell me about the lymphoedema treatment you received?

- What treatment did you have?
- What change did you notice? What difference did it make for you?
- What challenges did the treatment give you?
- Were you working when you developed lymphoedema?
- Were you looking after young children when you developed lymphoedema?

4. Expectations of treatment

What did you expect the treatment to achieve?

- What did you hope to achieve?
- What would have been the best result? What would be an acceptable result?
- What do you think needs to be done to get maximum improvement in lymphoedema?

5. Views of intensive treatment

Intensive decongestive lymphoedema treatment is offered to some women. This is a 2-3 week course of daily treatment with multi-layer compression bandaging replaced most days and worn day and night (*show pics as A4*), manual lymph drainage (a gentle lymphatic massage), exercise and skin care.

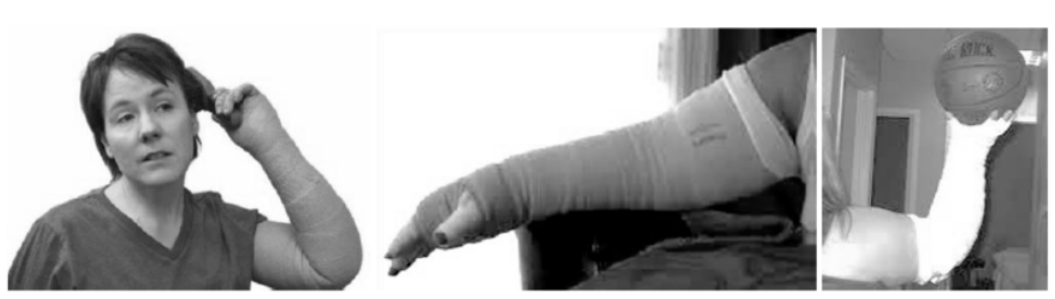

- What are your views on intensive treatment being offered to all women with lymphoedema?
- What are your views on intensive treatment being offered as the first treatment when you develop lymphoedema?
- What do you think the benefits would be?
- What would the challenges be for you?

6. Views on future treatment for women newly presenting with BCRL

We are planning a study to evaluate different lymphoedema treatments and identify the most effective treatment to be given to women when they first present with arm lymphoedema.

So, imagine that you have just developed arm swelling and are going to the lymphoedema clinic for your 1st appointment. What would you think or feel if they invited you to consider participating in a study comparing usual treatment (hosiery, exercise, skin care, self-massage) with standard care plus a 2-3 week course of intensive treatment? The computer would randomly allocate your treatment – you would not be able to decide which treatment you had. (*show A4 pics, usual care & intensive treatment, as above*)

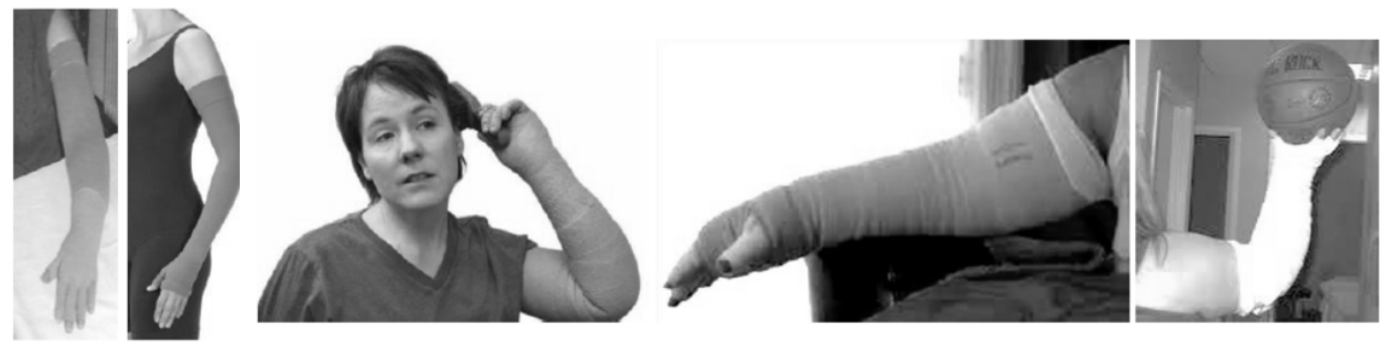

What are your views?

- Would you want to participate?
- What would be the challenges?
- What would make you want to participate in the study?
- If you would need to travel daily to the clinic for intensive treatment, would this be a problem?
- Would it make a difference if, instead of bandages you wore a compression sleeve and had 1 hour daily treatment with a compression pump? (*show A4 pics, pump and bandages*)

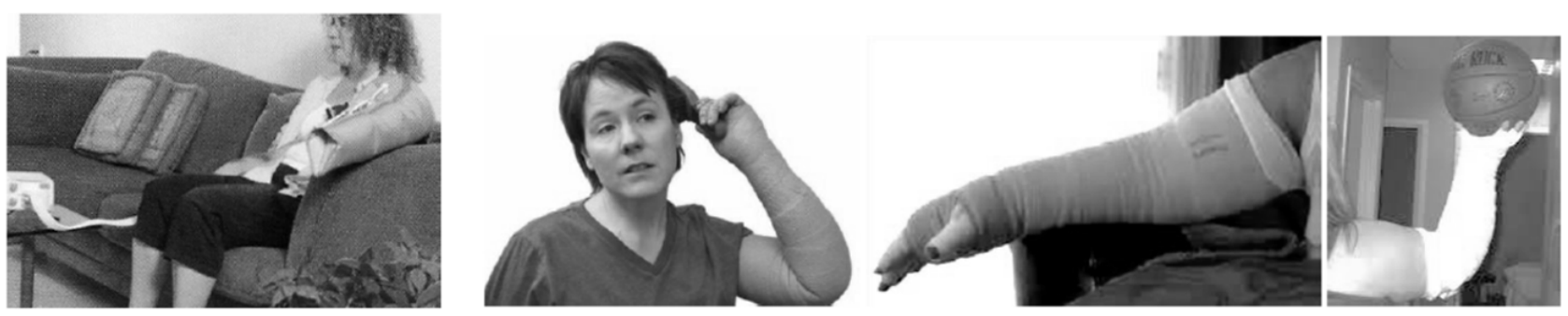

<u>7. Monitoring treatment outcomes</u>

How is your lymphoedema monitored?

HCP usually monitor size (volume) and quality of life. What else do you think should be monitored/measured?

<u>8. Patient-centred outcomes</u>

What is important to you? E.g. size, texture/feel of swelling, heaviness inside arm, pain, function/use of arm, other?

One tool asks patients to identify 3 key issues/problems relating to their lymphoedema that they would like to address. The severity of the problem is rated at each visit to monitor progress. The measurement is personal and individual to that person, so cannot be compared with the progress of others. What do you think of this method? Would it help you to monitor your progress?

<u>9. Final thoughts</u>

Is there anything else you want to mention that hasn't been covered in the discussion?

Do you have any other suggestions for improving the treatment offered to women when they first present with lymphoedema? Or other ways to monitor progress?

Thank you for your contribution. What we have discussed today is confidential and your contribution will be anonymous in any published report of this study. So please remember that today's conversation stays between us. Please let me know if you would like a copy of the final report although it may not be published until the whole project is completed in 2017.

Thank you again, and goodbye.

(b) Exemplar focus group topic guide for practitioners

(questions to be adapted according to group and, where appropriate, amended as the focus groups progress)

1. Introduction – of research team members and the format of the event, rules of group, etc.

2. Background
Can you tell me:

- How long have you worked with BCRL?
- Do you offer intensive lymphoedema treatment or only self-management with hosiery?
- Are you based in a hospital clinic, hospice or community clinic?

3. Experiences of lymphoedema treatment
Thinking generally about the 2 phases of decongestive lymphoedema treatment (DLT) and what your clinic offers, can you first describe the intensive treatment programme you offer?

- What individual components of treatment are included?
- Are you using standard MLLB or Coban2 system?
- How long does a treatment course last? Does the length of course vary at all?
- How do you decide whether to offer a woman DLT?
- What do you expect this treatment to achieve?
- What happens when the intensive treatment phase comes to an end?

Thinking now about the self-care phase, what does your maintenance phase look like?

- What individual components of treatment are included?
- How often would you see the patient?
- What do you expect this treatment phase to achieve?

4. Current care for women presenting with BCRL of the arm
Moving on to think specifically about treating women with BCRL of the arm,

- What treatment could a woman newly presenting with BCRL typically expect to receive?
- What effect/benefit would you expect the treatment to have?

5. Optimal lymphoedema treatment
Thinking about treatment benefit,

- What do you think is the most effective/ the optimal treatment to give to women newly presenting with arm lymphoedema? What do you think needs to be done to get maximum improvement in lymphoedema?
- What do you think could be achieved with optimal treatment?
- What would your ideal treatment programme look like? What individual elements of treatment would be included?
- What are your thoughts on what patients consider the optimal/most effective treatment to be?

6. Views of intensive treatment
In the UK, intensive decongestive lymphoedema treatment is offered to some but not all women. Thinking about the NICE recommendation that all women with BCRL are offered intensive treatment, that is phase 1 decongestive treatment, followed by maintenance treatment (phase 2),

- What are your views on intensive treatment being offered to all women with BCRL?

- What are your views on intensive treatment being offered as 1st treatment for BCRL?
- What do you think the benefits would be?
- What would the challenges be for your clinic?

7. Monitoring treatment outcomes

Thinking more about the impact of treatment, how do you monitor or measure lymphoedema treatment outcomes?

- What else do you think should be monitored/measured? What additional things would you like to monitor or measure if you had time and appropriate measurement tools?
- What outcomes do you think are important to the patient? *(size, appearance, texture, sensations)*
- One assessment tool asks patients to identify 1-2 key issues/problems relating to their lymphoedema that they would like to address. The severity of the problem is rated at each visit to monitor progress. The measurement is personal and individual to that person, so cannot be compared with the progress of others. What do you think of this method? Would it help you to monitor progress?

8. Views on future RCT for women newly presenting with BCRL

We think that a more intensive form of treatment may improve the long-term benefits and cost of treatment. We are planning a 6 month study to identify the most effective treatment to be given to women when they first present with arm lymphoedema.

What are your views on a randomised controlled trial comparing standard care with 2-3 weeks intensive decongestive treatment plus standard care (maintenance phase)? The plan is have 2 interventions, so patients would be randomised to one of 3 groups: A=intensive phase is multi-layer bandaging & MLD followed by standard maintenance phase treatment; B=PCP & hosiery & MLD followed by standard maintenance phase treatment; C= standard maintenance phase treatment only.

- Do you think patients would want to participate in such a trial as their first BCRL treatment?
- How should we recruit? Letter & information with 1st appt letter
- What would be the challenges for clinical staff?
- Do you think it will make a difference clinically or to patient tolerance to have 2-3 weeks intensive treatment?
- What are your thoughts on the 2 different interventions? A= multi-layer bandaging & MLD; B=PCP & hosiery & MLD
- What are your thoughts regarding standard MLLB versus the Coban-2 system?

9. Commissioning lymphoedema services

Do you participate in the commissioning process?

Do you commissioners understand what is needed for women with BCRL?

10. Final thoughts

Is there anything else you want to mention that hasn't been covered in the discussion?

Do you have any other suggestions for improving the treatment offered to women when they first present with lymphoedema? Or other ways to monitor progress?

Thank you for your contribution. What we have discussed today is confidential and your contribution will be anonymous in any published report of this study. So please remember that today's conversation stays between us. Please let me know if you would like a copy of the final report although it may not be published until the whole project is completed in 2017.

Title: Women's and practitioners' views of early intensive decongestive lymphoedema treatment for breast cancer-related arm lymphoedema: a focus group study.

Journal of Cancer Survivorship.

Authors: Jeffs E, Ream E, Chang YS, Taylor C, Bick D. corresponding author: Jeffs E. King's College London and Guy's & St Thomas' NHS Foundation Trust. Eunice.jeffs@kcl.ac.uk; Tel: 07557325642
