# Supplementary material for: Women’s and practitioners’ views of early intensive decongestive lymphoedema treatment for breast cancer-related arm lymphoedema: a focus group study
Source: J Cancer Surviv. 2025 Dec 2;20(5):2460–73. doi: 10.1007/s11764-025-01897-z (PMC13723537; doi:10.1007/s11764-025-01897-z)
Supplement: Supplementary file 4 [file 11764_2025_1897_MOESM4_ESM.pdf]

Supplementary Information 4: Initial thematic framework for women and practitioner interviews

Women's themes		Practitioner themes	
**1**	**Treatment**	**1**	**Treatment**
1.1	Usual care	1.1	Usual lymphoedema treatment
1.2	Variations to usual care	1.2	Usual care for new patients
1.3	Treatment goals	1.3	Variation to treatment
1.4	Cost of treatment	1.4	Role of DLT
1.5	Optimal treatment	1.5	Criteria for intensive treatment
1.6	Treatment not given/received	1.6	Additional/ideal BCRL treatment
1.7	Support	1.7	Challenges with treatment
1.8	Self-sourcing treatment	1.8	Self-sourcing treatment
1.9	Hosiery provision	1.9	FP10 hosiery
1.10	Hosiery challenges	1.10	Hosiery challenges
1.11	Previous lymphoedema treatment	1.11	Previous treatment
1.12	Re-referral for treatment	1.12	Changes to treatment approach
		1.13	Monitoring
		1.14	Perceived value of treatment
		1.15	Perception of treatment
		1.16	Waiting time for treatment
		1.17	Decision-making process
		1.18	Expectations of treatment
**2**	**Assessment**	**2**	**Assessment**
2.1	Methods	2.1	Priority methods
2.2	Value/ranking of forms	2.2	Value of assessment forms
2.3	Feasibility of assessment forms	2.3	Acceptability of assessment forms
2.4	Views on LSIDS-A	2.4	Views on LSIDS-A
2.5	Views on QuickDASH	2.5	Views on QuickDASH
2.6	Views on SATISIDS	2.6	Views on SATISIDS
2.7	Perceived lymphoedema severity	2.7	Additional things to monitor
2.8	Priority outcomes	2.8	Feasibility
		2.9	Challenges
		2.10	Complex BCRL presentations
**3**	**Study participation**	**3**	**Study issues**
3.1	Recruitment challenges	3.1	Acceptability of study
3.2	Acceptability of study	3.2	Acceptability of interventions
3.3	Acceptability of forms for study	3.3	Alternative interventions
3.4	Reasons for doing research	3.4	Capacity issues
3.5	Acceptability of intervention	3.5	Practitioner forms
		3.6	Practitioner screening patients
		3.7	Potential confounders
		3.8	Recruitment challenges
4	***Challenges with treatment***	***4***	***Patient education***
4.1	Getting tired	4.1	Materials used
4.2	Impact of delay obtaining treatment	4.2	Exercise instruction
4.3	Hosiery worn out/not enough supply	4.3	SLD instruction
4.4	Resource management	4.4	The (un)informed patient
4.5	Lack of knowledge/information	4.5	Information overload
		4.6	Breaking bad news
5	***Information***	***5***	***Treatment goals***
5.1	Access to information	5.1	Exercise programme
5.2	HCP lack of knowledge	5.2	For maintenance phase
5.3	Not aware if …?	5.3	For intensive treatment phase

6	***Treatment outcomes***	***6***	***Organising care***
6.1	Don't know what to expect	6.1	Adapting treatment
6.2	High hopes of a cure	6.2	Change to service provision
		6.3	Planning discharge
		6.4	Managing caseload
		6.5	Managing/access to return to lymphoedema service
		6.6	Intensive treatment for all lymphoedema patients
		6.7	Different practitioner approaches to delivering care/instruction
7	***Symptoms experienced***	***7***	***Uncertainties of treatment***
7.1	Physical symptoms: size,	7.1	Uncertainty re treatment workings
7.2	Constant awareness of swollen arm	7.2	Uncertainty re what treatment is needed
7.3	Limitations of symptoms	7.3	Uncertainty re expectations of treatment (possible outcomes)
7.4	Understanding interplay between symptoms/health problems		
7.5	Pins & needles		
8	***Feelings about treatment***	***8***	***Women newly presenting with BCRL***
8.1	Living with lymphoedema	8.1	Typical clinical characteristics
8.2	Feel naked without sleeve on	8.2	What treatment do women want?
8.3	Cautious about what I can and can't safely do	8.3	What treatment they are offered
8.4	Interest in being involved with research	8.4	Expected treatment outcomes
8.5	At least I'm alive	8.5	Likely duration within lymphoedema service
8.6	this treatment is as good as it gets	8.6	Optimal treatment for BCRL
8.7	Staff approach makes a difference		
9	***Managing lymphoedema***	***9***	***Patient issues***
9.1	Lymphoedema and its treatment not always priority	9.1	Concerns re discharge
9.2	Family support	9.2	Shock/surprise at lymphoedema diagnosis/treatment
9.3	Exchanging tips for lymphoedema care/life		
		10	***Future RCT for women newly presenting with BCRL***
		10.1	Recruitment
		10.2	Burden of treatment
		10.3	Measuring outcomes
		10.4	Minimal clinically importance difference?
		10.5	Challenges for lymphoedema practitioner
		10.6	Components of intervention package

Note: [1]Analysis conducted in 2016

Title: Women's and practitioners' views of early intensive decongestive lymphoedema treatment for breast cancer-related arm lymphoedema: a focus group study.

Journal of Cancer Survivorship.

Authors: Jeffs E, Ream E, Chang YS, Taylor C, Bick D. corresponding author: Jeffs E. King's College London and Guy's & St Thomas' NHS Foundation Trust. Eunice.jeffs@kcl.ac.uk; Tel: 07557325642
