# Supplementary material for: Women’s and practitioners’ views of early intensive decongestive lymphoedema treatment for breast cancer-related arm lymphoedema: a focus group study
Source: J Cancer Surviv. 2025 Dec 2;20(5):2460–73. doi: 10.1007/s11764-025-01897-z (PMC13723537; doi:10.1007/s11764-025-01897-z)
Supplement: Supplementary file 5 [file 11764_2025_1897_MOESM5_ESM.pdf]

Supplementary Information 5: Matrices for (a) women and (b) practitioners
(reported as (1) group-level analysis and (2) individual contributions)

(a) Matrices for women, focus group 1 (FGW1) and focus group 2 (FGW2)

<u>Theme 1: 'Usual' care for women with BCRL</u>					
**Case name**	**1.1 Current lymphoedema treatment**	**1.2 Variations to lymphoedema treatment**	**1.3 What is treatment?**	**1.4 Adherence to recommended lymphoedema treatment**	**1.5 Burden of self-treatment, 'usual' care**
**Focus group 1** (FGW1)[1]	Usual care was hosiery and exercise; skin care only mentioned by one woman although possible all women did skin care but don't consider it to be treatment. All women wore hosiery and 4 (out of 5) mentioned exercise.	Treatment changes made to deal with fluctuations in swelling and find better control of swelling. Most reported variations were to compression garments; main change was a move to MTM[2] garments, particularly when arm too large for OTS[2] hosiery.<br>Only one woman had previously received a course of phase 1 DLT, to deal with more severe swelling after years of self-treatment.	[n.d.]	Women report being generally concordant: following instructions re garment wear and exercises	Women had difficulty obtaining hosiery on prescription because neither GP nor pharmacist knew what to do.<br>Visibility of hosiery acted as a sign of lymphoedema and caused people to ask questions<br>Limited number of garments allowed per year caused problems with hygiene, particularly with hand oedema

[1] FGW1 included 5 women with BCRL of 2-10 yr duration, all doing self-treatment; only one had received phase-1 DLT. One woman had bilateral arm & hand oedema, one had breast oedema. They were aged 40-75 years, 4 were white British and 1 black British. Only 1 woman was responsible for pre-school/school age children at referral for treatment
[2] MTM=made-to-measure; OTS = off the shelf

Theme 1 (cont): 'Usual' care for women with BCRL					
**Case name**	**1.1 Current lymphoedema treatment**	**1.2 Variations to lymphoedema treatment**	**1.3 What is treatment?**	**1.4 Adherence to recommended lymphoedema treatment**	**1.5 Burden of self-treatment, 'usual' care**
**Focus group 2** (FGW2)[1]	All wore hosiery. Some women did exercise, and some also did SLD. Only three women had previously had a course of intensive treatment: one had several courses of DLT followed by modified DLT with pump and MLD; one had a course of modified DLT with pump and MLD; one had a course of 15 MLD treatments.	Most women had variations only to hosiery, to manage fluctuation in symptoms and gain better control of swelling.<br>One woman had previously received a course of DLT to deal with more severe swelling and recurrent cellulitis after years of self-Rx. Another woman received a course of modified DLT with pump and MLD to improve arm symptoms.<br>Another woman started lymphoedema treatment with 15 privately funded MLD treatments (all that was allowed by her insurance company)	Women thought practitioner-delivered treatments were more effective than hosiery, including those who had received them. Some women questioned whether self-treatment improved symptoms.	Women were generally concordant, although variation within group particularly re hosiery wear time. Some women wore hosiery all the time, others only wore hosiery or increased their wear time when they noticed worsened symptoms	Women were frustrated by the lack of HCP knowledge or conflicting information supplied.<br>They had difficulty getting hosiery via GP and local pharmacy.<br>Visibility of hosiery triggered unwanted questions from the public – women didn't know how best to always handle these questions.

[1] FGW2 included 8 women with BCRL of 6m-12 yr duration and all doing self-treatment; only one had received phase-1 DLT and 2 had modified DLT. One woman also had breast oedema. Women were aged 40-75 years and all were white British. Only 1 woman was responsible for pre-school/school age children at referral for treatment

[2] SLD=self-lymphatic drainage; DLT=phase-1 DLT; MLD=manual lymph drainage

Theme 2: Monitoring treatment outcomes				
**Case name**	**2.1 Assessment of lymphoedema**	**2.2 Challenges for monitoring**	**2.3 Perceived lymphoedema treatment outcomes**	**2.4 Priority outcomes**
**Focus group 1** (FGW1)	[n.d.]	Women highlighted their difficulty in deciding how to answer questions regarding function, whether to answer according to what they can do (regardless of the consequences of the task) or what they might choose to do.<br>They suggested women could benefit from examples to help them identify their personal issues and the impact of lymphoedema, that they would need time following a lymphoedema diagnosis to identify personal issues to be monitored.	All had seen some improvement with self-treatment. One had received DLT with good benefit the first time and less benefit from the 2nd course.	All agreed re importance of monitoring size and issues of personal importance. They did not offer examples of issues important to them but talked in general terms of what might be important e.g. appearance, function, any pain
**Focus group 2** (FGW1)	Little mention and no group discussion about current methods of assessment, although these were alluded to when taking about challenges of monitoring and priority outcomes.<br>They felt that regular informed self-monitoring would improve awareness of need for self-treatment, but guidance was needed to make sense of information.	Group discussed challenges of completing the QOL form, particularly where non-lymphoedema issues impacting QOL and when lymphoedema is not their priority.<br>They agreed with W13 that it was important to have a conversation about the impact of treatment, as well as lymphoedema, and highlighted the value they placed on regular monitoring to support, motivate, as well as make sense of changes to symptoms.	Agreement that their symptoms had improved with self-treatment, although most mentioned stability or control of symptoms. Those who had experienced practitioner-delivered treatment reported greater benefit than with self-treatment alone.	Group all felt it was important to measure size. They identified five aspects for monitoring: size, appearance, personalised issues, body image perception, and finding out how I am getting on.

Theme 3: Acceptability of proposed study			
**Case name**	**3.1 Acceptability of participation in future lymphoedema research**	**3.2 Burden of proposed study interventions**	**3.3 Treating early-BCRL with intensive-DLT - is it worth it?**
**Focus group 1** (FGW1)	Group had varied understanding of trials and randomisation. Group opinion was they would have opted for participating in the trial as they all considered their swelling to be bad enough at the start. They thought women would participate because they wanted to help other women in years to come; that women would think the study invitation meant practitioners thought the study treatment was likely to work. Comprehensive information about lymphoedema and treatment options was needed to decide whether to participate.	The one person who had experienced DLT shared her experience with the group. Women were concerned about limitations to daily life when wearing bulky bandages. They did not think daily travel was a problem as women were used to daily cancer treatment<br>Group agreed modified DLT would be more acceptable, i.e. replace bandages with pump & hosiery.	Group discussed how acceptability and value of DLT was influenced by their experience of lymphoedema treatment and having come to terms with lymphoedema. They recognised they would not have had this knowledge at start of treatment and would have been willing to gamble several weeks on early DLT to kick-start a reduction in swelling. They concluded DLT was only worth doing if patients perceived swelling as 'bad enough' to justify it.<br>Study timing may be problem if only recently completed Cancer Rx and didn't want intensive treatment.
**Focus group 2** (FGW1)	Not discussed. The group focused on whether they would be willing to have early DLT and potential timing of the study rather than willingness to participate in research. Re timing, they suggested avoiding the immediate period post cancer treatment.	Group agreed there would be challenges with the bandaging element of DLT, particularly if working or lived alone.<br>Modified DLT was proposed by a group members as more acceptable and less intrusive than bandages. Group all agreed that daily treatment with pump and sleeves was more acceptable than bandages as no restriction outside of the treatment visit.	Group had mixed views on whether they would have opted for early DLT. They wanted to know they had done everything possible to reduce lymphoedema symptoms, but were not sure early DLT with bandaging would provide sufficient benefit to justify the inconvenience and effort required.

Theme 4: Living with lymphoedema				
**Case name**	**4.1 Physical and psychological consequences of BCRL**	**4.2 Learning to live with lymphoedema**	**4.3 Hopes, expectations and beliefs about lymphoedema treatment**	**4.4 Impact of knowledge deficit**
**Focus group 1** (FGW1)	Impact of swelling includes: not able to wear jewellery, difficulty with fit of clothes, fluctuation in swelling, fear of deterioration if lift things.	Made choice to learn to live with and accept lymphoedema, to ask for help with chores to minimise impact on lymphoedema.<br>Other life circumstances may take priority, e.g. ill health in the family; cancer treatment	Group discussed lack of information about lymphoedema, treatment options and what outcomes they could expect.<br>They agreed with Researcher suggestion that goals focused on accessing treatment rather than treatment outcomes.<br>Group agreed they accept their current level of progress and can't see how things can improve.	Lack of information about lymphoedema, so wary of impact of heavy lifting on arm swelling.<br>Challenges with pharmacist and GP
**Focus group 2** (FGW1)	Range of ways BCRL impacts them.<br>Mixed views re impact of BCRL as reminder of cancer; most agreed the visibility of lymphoedema and hosiery triggers unwanted questions which lead to mention of cancer; some think BCRL can make it difficult to move on from cancer.<br>Group agreed BCRL and the visibility of hosiery create significant body image issues	Mostly women try to get on with life: some carry on life as before, others are wary of consequences to swelling to ask for help or avoid activities for fear of exacerbating swelling.	Group agreed they didn't know what to expect from treatment, but believed there was hope of improvement and better control. Treatment outcomes are not assured, and they question who knows what is achievable.	Group were frustrated by their personal lack of knowledge about lymphoedema, its treatment and impact on life.<br>They questioned the level of knowledge amongst HCPs, and whether lymphoedema practitioners were up-to-date re treatment advances.

Theme 1 (women's individual responses): 'Usual' care for women with BCRL (FGW1)					
**Case name[1]**	**1.1 Current lymphoedema treatment**	**1.2 Variations to lymphoedema treatment**	**1.3 What is treatment?**	**1.4 Adherence to recommended lymphoedema treatment**	**1.5 Burden of self-treatment, 'usual' care**
W1	Hosiery; exercise; booklet; reviewed every 6 months Sleep in glove to control hand swelling.	Tried many different garments: to find garments which didn't cause skin reaction; to thicker garments to better control swelling.	[n.d.]	Always wore garment because uncomfortable without it. Tried to do exercise but difficult as tiring & can be painful.	Had to wait 6m for hosiery: GP couldn't find computer prescription codes; Pharmacist didn't know how to order hosiery. Hosiery worn out before due for replacement. *"Each time I asked for a prescription, my GP said they cannot find it, they can't prescribe it. The only female doctor, she researched and researched to be able to get the right prescription for me. And when I took it to the pharmacist, nothing, I don't get it…I stood there for like an hour, she was on her computer. She could not get the right prescription."* Visibility of hosiery triggered unwanted questions. *"It's so awful most times, like when I walk in at school time, the kids, "Oh Miss, why have you got this on?" The questions, all the time."* Pins & needles worse when wore sleeve.
W2	MTM hosiery; exercise; booklet/info	Tried various garments. Arm too big for OTS garment – sleeve welt cutting into arm & rolling down – so now has custom garments	[n.d.]	Always wore garment, except for special occasions – felt naked if not wearing sleeve. Did exercise. Trusted HCPs and did what they say.	Positive experience getting FP10 hosiery. Agreed with W1 & W4 re visibility of hosiery, causing public questions Hesitant about what activities she can and can't do. Lymphoedema is not always her priority *"When I was first diagnosed three years ago, I also had my daughter at home with bowel cancer. So I didn't only have all this to contend with"* *Res: "So lymphedema was probably not your priority at the time?"* *"No, no at the time nothing was. (…) So I just wear my sleeve every day and that's it."*

[1]For participant characteristics, see Table 3a

Theme 1 (cont): 'Usual' care for women with BCRL (FGW1)					
**Case name**	**1.1 Current lymphoedema treatment**	**1.2 Variations to lymphoedema treatment**	**1.3 What is treatment?**	**1.4 Adherence to recommended lymphoedema treatment**	**1.5 Burden of self-treatment, 'usual' care**
W3	MTM hosiery	Arm too big for OTS garment so now wears MTM garments	[n.d.]	Sleeve worn daily waking hours	Problems obtaining FP10 hosiery. Agreed with W1 & W4 re visibility of hosiery, causing public questions. Lack of HCP knowledge re causes of hand swelling and recognising cellulitis
W4	Hosiery; exercise	Initially, resolution of swelling, so stopped wearing sleeve.<br>Several years later had a new episode of swelling so recommenced treatment	[n.d.]	Doing exercise & being good about wearing sleeve	3-month delay receiving treatment may have affected outcome. Wanted more information about lymphoedema and treatment options when first referred. Hosiery very visible, so people ask questions. Also difficulty getting hosiery. Wary of possible impact of heavy chores, so asked family to help.<br>*"You have hit a point with me in that I'm sure we're all in the same boat where you're always getting people in shops or wherever saying, "Oh what have you done to your hand?""*
W5	MTM hosiery, exercise, skin care; reviewed every 6-12m. Takes painkillers.	Had lots of different garments and now wears custom sleeve for best fit. Previously had 2 lots of bandaging to reduce severity of lymphoedema, plus did SLD. Felt she had lots of treatment choices, including course of DLT, but *"initially…it was a little trial and error"*	[n.d.]	Does what she is asked to do. Has looked after arm.	Mentioned no problems with current treatment.<br>Talked about previous experience with intensive treatment and trial and error to find sleeves that worked.<br>*"I suppose the travelling, the putting up with it, the wearing of clothes and, well, the sessions at the clinic are long enough for them to undo them because the bandaging is very, very long."*

Theme 1 (cont): 'Usual' care for women with BCRL (FGW2)					
**Case name**	**1.1 Current lymphoedema treatment**	**1.2 Variations to lymphoedema treatment**	**1.3 What is treatment?**	**1.4 Adherence to recommended lymphoedema treatment**	**1.5 Burden of self-treatment, 'usual' care**
W6	Hosiery – sleeve and glove for 5 years	No variation in treatment, only different colour sleeve	[n.d.]	[n.d.]	Can't wear light coloured sleeves as the 'go rotten'.<br>Difficult to get black sleeves<br>Lymphoedema is not a priority:<br>*"My biggest problem is I had the 18 months of Herceptin and I went from ten stone to 12 stone, and I can't shift it. That is my biggest problem. My arm's not."*
W7	Hosiery; exercise. Reviewed annually	Change to type of sleeve.<br>Also had 15 sessions of MLD paid for by health insurance & more MLD from BC charity	[n.d.]	Sleeve worn only when being really good.<br>Difficult to wear hosiery in hot weather	Not sure the HCP fully understand the impact of LO. Told by GP to hold hand in air to treat lymphoedema.<br>Hosiery visible so people ask questions. Is important to have comfortable hosiery but it needs to also look good.

Theme 1 (cont): 'Usual' care for women with BCRL (FGW2)

Case name	1.1 Current lymphoedema treatment	1.2 Variations to lymphoedema treatment	1.3 What is treatment?	1.4 Adherence to recommended lymphoedema treatment	1.5 Burden of self-treatment, 'usual' care
W8	Hosiery – sleeve & gauntlet<br>Massage (?SLD), skin care	Hosiery same type as original garments.<br>Chose to stop wearing sleeve regularly as thought swelling had improved sufficiently. But as lymphoedema clinic reported increased swelling at last appointment, recommenced wearing hosiery.	*"I'm not even clear what the self-care is. But people talk about exercise; just the exercise – is that self-care?"*	Currently wearing sleeve regularly as arm worse, but wasn't wearing it a month ago. Expects to slip again so needs to be pragmatic about treatment. Has done massage.<br>*"I'm sort of in the opposite position at the moment, having been assiduous with the old massage and creams and wearing the sleeve for a whole year, and I thought that was good. Fine, I'll not wear it so much. Went back (to clinic) about a month ago feeling fine, hadn't hardly been wearing it at all – it's got a lot worse. ((Laughs)) Put (sleeve) back on all the time! In some way I knew it wasn't right but I kind of kidded myself I was fine."*	Only get 2 sets of hosiery per year, so get filthy and torn. Works as jeweller, so 2 sets of garments is not enough.<br>Hosiery visible so people ask questions.
W9	Hosiery	Hosiery varied - needed handpiece<br>When swelling worsened, was given modified DLT (pump & MLD). Now hand swelling has improved, only wear armsleeve.	[n.d.]	[n.d.]	Consultant ignorant of prevalence of lymphoedema & fluctuations in swelling.<br>HCPs focus on need to avoid insect bites but ignorant about impact on lymphoedema of carrying loads.<br>You're on your own after discharge.

Theme 1 (cont): 'Usual' care for women with BCRL (FGW2)					
**Case name**	**1.1 Current lymphoedema treatment**	**1.2 Variations to lymphoedema treatment**	**1.3 What is treatment?**	**1.4 Adherence to recommended lymphoedema treatment**	**1.5 Burden of self-treatment, 'usual' care**
W10	Hosiery	Initially no treatment, then several types of sleeve when LO did not spontaneously resolve. Privately funded some MLD, but not suitable as exacerbated another health condition.	[n.d.]	Can't wear hosiery in hot weather due to eczema. Now more adventurous and adapted self treatment by reduced hosiery wear time and increased use of arm. *"I'm becoming a little bit more adventurous now with (when I'm) leaving the sleeve on and picking up my granddaughter and stuff like that without thinking the arm will get worse. So, maybe it has stabilised."*	Had been told can only have 2 sleeves replaced every 12 months
W11	Hosiery; massage (?means SLD) to breast oedema; devised own exercise prog; booked for clinic education/ information session	Self-sourced information and treatment (MLD). [*Has only first assessment visit, and yet to have follow-up visit, so no treatment variation to date*]	Doesn't think she has treatment: *"Not after this conversation, because you were saying there was a decongestive stage and a maintenance stage, and the sleeve is the maintenance stage. I feel like I've just been given the maintenance stage right at the very start." "The conversation wasn't even taken up that there are other treatments."*	[n.d.]	Lack of HCP knowledge re local lymphoedema incidence. Lymphoedema clinic unaware of new surgical techniques. *"I was disappointed because I knew more than HCP did."* Agreed with W7 re visibility of hosiery and hating questions that arise.

Theme 1 (cont): 'Usual' care for women with BCRL (FGW2)					
**Case name**	**1.1 Current lymphoedema treatment**	**1.2 Variations to lymphoedema treatment**	**1.3 What is treatment?**	**1.4 Adherence to recommended lymphoedema treatment**	**1.5 Burden of self-treatment, 'usual' care**
W12	Hosiery; exercise "doing self care" (p5)	Tried various garments, light weight and flat knit. DLT recommended when nothing else was working.<br>Had several courses of DLT, and lots of sessions of bandaging, pump and MLD. Pump used to control fluctuations in swelling.<br>Long-term antibiotics for recurrent cellulitis	*"I think when I've had the bigger interventions, as it were, I felt at least I'm doing something. And so that is a psychological thing: this could help. Whereas just the sleeve thing doesn't feel sometimes quite enough. You want more. Ideally you want a pill, don't you?"*	Used to wear sleeve only part of the day. Now wear all day and at weekend. Prefer to wear it to control swelling, even in hot weather.<br>*"(At first) I would probably not wear (the sleeve) a whole day. Whereas now I can wear it all day because it does swell up more in the heat. Therefore, I prefer to wear it even when it's hot."*	Agreed with others re visibility of hosiery triggering unwanted questions. Didn't always know how best to answer questions.
W13	Hosiery – sleeve & glove (altho also referred to hosiery as 'bandages'); exercise	Has had changes in type of hosiery. Increased wear time for hosiery. Swelling worsened, especially hand, so self-adapted treatment.<br>Self-adjusts treatment according to perceived severity of symptoms	[n.d.]	Initially wore hosiery only when exercising. When swelling got worse, started wearing hosiery all the time. Now diligent doing exercises and wearing gloves when typing.	Frustrated by lack of knowledge among HCPs and conflicting advice, e.g. re exercise and activities, amount of weight that can safely be carried, likely outcome of treatment. Practitioners give a bleak prognosis, when truth is nobody actually knows.<br>Considers hosiery to be the colour of sickness. Agreed with others re visibility of hosiery triggering unwanted questions.<br>*"That colour just is the colour of illness. Nobody ever wears anything that colour unless there's something wrong with them. ((group laughter)) And actually it's like advertising, 'I'm a bit sick'."*

Theme 2 (women's individual responses): Monitoring treatment outcomes (FGW1)				
**Case name**	**2.1 Assessment of lymphoedema**	**2.2 Challenges for monitoring**	**2.3 Perceived lymphoedema treatment outcomes**	**2.4 Priority outcomes**
W1	Size	Nowhere on form to explain consequences of doing things, e.g. *"I still try (to do things), but I end up with pains and swollen arm."* *"I don't do as much as I used to do. (…) Whenever you go to the clinic there is this survey about how you feel and you rate. But there is nowhere for you to explain. Yeah of course we do things, but you get tired easily and you don't end up finishing what you've started."* *"It's tricky, but my girls, one is ten and 12 year, they are always there to help. (…) So like I share the job, what I know I cannot do, they do. They push the trolley and things like that."*	Left hand fluctuates but right hand not reducing. Implied sleeve reduced arm heaviness and increased comfort	Measurements.<br>Arm heaviness
W2	Monitors size at wrist (circling wrist)	Agreed with W4 that difficult to monitor function due to potential consequences of doing an activity, "*You're hesitant about what you can do and what you can't do. I think so."*	Arm did go down.	Size. Agreed it was also important to monitor issues of personal importance
W3	Monitors size, also impact of chores/activities on size	Agreed with W4 that need prompts to help identify personalised goals and issues to monitor	Was told at recent clinic appointment that arm had reduced.	Size. Agreed it was also important to monitor issues of personal importance

Theme 2 (cont): Monitoring treatment outcomes (FGW1)				
**Case name**	**2.1 Assessment of lymphoedema**	**2.2 Challenges for monitoring**	**2.3 Perceived lymphoedema treatment outcomes**	**2.4 Priority outcomes**
W4	Monitor appearance and tightness of clothes	Difficult answering some questions as what can do and actually done, as not always the same. Unsure if due to BCRL or other health issue. *"I think it's difficult because we tend to be wary of the heavy shopping bag or moving pots outside my door. I usually get my husband in to do it. (…) So I think what you can do is a tricky one because we are naturally hesitant about going down that route and doing the ironing or whatever."* Thought new patients might need more time to identify lymphoedema-specific issues, e.g. heaviness, pain. Agreed with W5 re receiving forms prior to appointment with time to identify issues for completion at clinic. *"I think you might need to give examples of, for example, pains, heaviness of arm or whatever, if you were new to lymphedema and not really quite sure."*	Swelling completely resolved (1st episode) with sleeve. 2nd episode: swelling now stable when wearing sleeve.	Should monitor issues of personal importance, e.g. appearance & tightness of clothes, whether have pain, function.
W5	Monitors appearance of arm	Monitoring needs to be personalised. People need time to think about how lymphoedema impacts them, so may be useful to send out questionnaires before appointment and give options to think about when completing the form. Women may better understand how they feel if assessed after 6 months of BCRL treatment. General well-being can vary according to all sorts of things, not just lymphoedema. *"We're all individuals…we've all differences (…) I think a personalised sheet, I know it's a lot of work for nurses, but a personalised sheet seems very good."*	Took long while for initial swelling to reduce. 1st course of DLT reduced arm to nearly normal ('skinny again'). 2nd course less successful as swelling increased after bandaging but reduced with sleeve. Swelling very stable at present. *"I think we perhaps accept at the moment the level to which the treatment has progressed…I've personally got to the level that I believe is possible for me in as much as I've got the right garment and I am still being seen every 12 months or with contact at any time available to me."*	Should monitor personally relevant issues, e.g. appearance. *"I know it's a lot of work for nurses, but a personalised sheet seems very good."*

Theme 2 (cont): Monitoring treatment outcomes (FGW2)				
**Case name**	**2.1 Assessment of lymphoedema**	**2.2 Challenges for monitoring**	**2.3 Perceived lymphoedema treatment outcomes**	**2.4 Priority outcomes**
W6	Was told could go to the clinic if problem, but not needed to	*"I don't want to particularly (go to the clinic), not unless I have to"*	[n.d.]	None stated as main concern was not lymphoedema but sustained weight gain with Herceptin
W7	It is important to have a point of regular contact with clinic, especially when feeling vulnerable	Monitoring appointments can be used to check info retained – easy to lose information, or can be too much to take in at once	Helped by using trampette and exercise.<br>Found arm much better after holiday in hot country & not wearing sleeve.	Mind, psychological impact, impact on femininity, appearance/body image
W8	*"They sound so sensible. I'd love that. I would really understand what was happening; whereas sometimes I don't. (…) I'm sure if I'd done that and done that regularly I would be a lot more informed about lymphedema and how I should treat it."*	Agreed with W13 that what was needed was more conversation about how managing with lymphoedema rather than more forms to complete	[n.d.]	Personalised outcomes. Find out how I'm getting on. Agreed with W11 that monitoring body image and QOL can be difficult.<br>Agreed that important to monitor size, appearance, texture & sensations.
W9	Not discussed	Challenge of self-monitoring as generally discharged after 3-5 years, and waste of time going to GP.<br>Factors other than lymphoedema affect quality of life.	Definitely got better	Personalised outcomes. Agreed size, appearance, texture & sensations important
W10	Not discussed	No mention.	Nothing seemed to work for several years, then settled & now stable.	*"Just the size"*

Theme 2 (cont): Monitoring treatment outcomes (FGW2)				
**Case name**	**2.1 Assessment of lymphoedema**	**2.2 Challenges for monitoring**	**2.3 Perceived lymphoedema treatment outcomes**	**2.4 Priority outcomes**
W11	[n.d.]	No pre-surgery baseline measurements taken. *"Your quality of life can be good but your body image perception could still be quite low even at the same time"*	[n.d.]	Body image perception, separate to QOL
W12	Recognised value of regular review, particularly circumference measurements, to motivate to continue or increase self-treatment	Not sure answers to QOL are very useful as can't remember what said before to know if it is improved - completing QOL form can be so variable.	Initially, mild swelling stable for years with hosiery. Then increased after breast cancer recurrence and had multiple bouts of cellulitis. Several courses of DLT helped. Sessions of pump & MLD probably helped start real maintenance and stability.	Agreed with others re including body image perception, separately to considering QOL. Also think change to size and personalised outcomes are important.
W13	Size	Finds the QoL form 'horrendous'. Doesn't like completing questionnaires and would prefer a conversation with practitioner about how she is getting on and able to talk about difficulty wearing a sleeve. *"Just having a conversation with a human being about it."* Has difficulty knowing if ache in hand/arm is due to BCRL or arthritis.	It improved. As soon as put hosiery on, arm felt more comfortable; plus, felt better because had some control of swelling with hosiery and exercise. Swelling variable but currently stable - arm is reasonably good, but hand aches.	Agreed with others that it is better to monitor body image perception as well as QOL. Important to monitor size and sensations in arm, particularly ache. Values personalised outcomes - finding out how I'm getting on. *"It would be really helpful if the nurse who was measuring my arm said, 'How are you getting on? How are you finding wearing a sleeve?' And I'd say, 'Actually it's difficult at church'. I'm a vicar. The kids come up to me and ask me if I'm a Michael Jackson fan! (pointing to glove)"*

Theme 3 (women's individual responses): Acceptability of study (FGW1)			
**Case name**	**3.1 Acceptability of participation in future lymphoedema research**	**3.2 Burden of proposed study interventions**	**3.3 Treating early-BCRL with intensive-DLT - is it worth it?**
W1	She didn't want to be experimented on and questioned how computer could decide the correct treatment for her arm<br>Agreed with group that women needed relevant information in order to decide whether to participate.	Daily attendance for treatment would have been acceptable as she was very comfortable and used to attending the hospital.<br>Didn't comment on modified DLT.	Changed opinion during group discussion: initially said early DLT was not acceptable because now accepting treatment. Realised this was benefit of hindsight and that she would have been desperate enough to accept DLT if offered when first presenting for treatment, with no treatment knowledge.<br>DLT would be acceptable if it made a difference, but not if still had to wear hosiery after treatment.<br>"*Going through (intensive-DLT) for three weeks and ending up still wearing a sleeve, I wouldn't go for it. I'd rather stick to my sleeve.*"
W2	Would have accepted trial. Wants to help others in the future, even if it doesn't benefit her now.<br>Would have participated in trial at beginning of treatment in case it did help her.	Would do what was told to do for treatment.<br>Suggested practical solution for keeping arm dry when showering.<br>Agreed modified DLT would be more acceptable alternative to bandages.	Prepared to do more than current treatment. Expressed mixed views: agreed with W5 that it would be worth trying early DLT to kickstart a reduction of swelling; that she trusted HCPs advice and would wear bandages if told to do so; thought early DLT would be worth having only if symptoms were very bad, and believed her presenting symptoms were severe enough to have accepted early DLT if offered.
W3	Would have accepted trial. Stated she thought a lot of people would accept the trial, but did not give reasons for this	Mentioned only daily attendance, but agreed with W4 re used to attending hospital.<br>Murmured agreement that modified DLT was more acceptable alternative to bandages.	Prepared to do more than current treatment. Stated it would have been better to have DLT at the beginning of treatment, and murmured agreement with W5 comment that it was worth trying early DLT to kickstart the reduction.

Theme 3 (cont): Acceptability of proposed study (FGW1)			
**Case name**	**3.1 Acceptability of participation in future lymphoedema research**	**3.2 Burden of proposed study interventions**	**3.3 Treating early-BCRL with intensive-DLT - is it worth it?**
W4	Would have accepted trial. Appeared informed about trials and principle of randomisation.<br>Believed women would be likely to participate if given relevant information about study, including explanation of lymphoedema and available treatments, expected outcomes for all treatments as well as specific information about the possible outcome as well as burden of study intervention/s<br>*"I think at that point most people don't actually understand what lymphedema is and what the long-term implications of it are… I think to have all that information set out is only good practice"*	Daily travel was not considered a problem as used to travelling for DXT.<br>Agreed modified DLT would be acceptable alternative to bandages.	Prepared to do more than current treatment. Thought phase 1 DLT would be worth having if only to achieve better swelling control than with self-treatment, even if arm size not significantly smaller and hosiery still needed at the end of course of intensive-DLT<br>Speculated whether early DLT would reduce swelling so sleeve needed only to prevent further swelling.<br>"*But when you're saying you're going back to wearing a sleeve, presumably that's with a reduced swelling on your arm, and the sleeve is then only a safety precaution as it were. And I think I'd go for that.*"
W5	Women need information about lymphoedema and treatment options as well as study treatments to decide whether to participate.<br>Would have accepted trial. Appeared informed about trials and principle of randomisation.<br>*"If it was at the very beginning and you didn't know anything about what was likely to be the result of wearing sleeves or any of the treatments, I think you would probably accept that, because you would believe then that anything that the clinicians or nurses or whoever said is likely to work."*	Bandages bulky, like Michelin man, so difficult finding clothes to fit. In same bandages over weekend & difficulty keeping bandages dry in shower. Daily travel time, and would need time off work unless retired.<br>Agreed modified DLT would be acceptable alternative to bandages.	Had experience of phase 1 DLT. Thought both courses of treatment were worth having although first course was most effective. Feels having early DLT would mean you'd done all you can – it would be a good kick start to reduce swelling.<br>If recommended by HCP it is worth doing as likely to help.

Theme 3 (cont): Acceptability of proposed study (FGW2)			
**Case name**	**3.1 Acceptability of participation in future lymphoedema research**	**3.2 Burden of proposed study interventions**	**3.3 Treating early-BCRL with intensive-DLT - is it worth it?**
W6	[n.d.]	Lack of time. Wearing bandage all the time.<br>Modified DLT is more acceptable as wearing sleeves not bandages between pump treatments.<br>"*A pump you can just walk away from after your time is up, can't you? When you've been that's it. But the bandage you've got on all the time.*"	Wouldn't have DLT, doesn't think DLT is worth it. Had seen people bandaged and been grateful it wasn't her.
W7	[n.d.]	Would be difficult if couldn't drive.<br>Didn't comment on modified DLT.	Dreamed of winning the lottery and having the money to travel to Austria to have phase 1 DLT
W8	Might not be the right time if just completed cancer treatment	[n.d.] Didn't comment on burden of DLT or modified DLT.	Was totally averse to doing anything extra to treat lymphoedema
W9	Considered lymphoedema a side issue compared to cancer treatment and its side effects.	Difficult if still working. Was retired when presented for treatment so daily travel would not have been a problem.<br>Modified DLT more acceptable as treatment. She had received benefit from pump and MLD treatment.<br>"*It worked for me, but by that time I'd taken early retirement so the work factor wasn't an issue. So, coming every couple of days or whatever it was to have the pump and the MLD was okay. It's reversed the swelling in my hand and I'm really, really grateful for that.*"	Thought it was better to ration resources to ensure everyone in the UK had some treatment, with bandaging reserved for more severe cases.

Theme 3 (cont): Acceptability of proposed study (FGW2)			
**Case name**	**3.1 Acceptability of participation in future lymphoedema research**	**3.2 Burden of proposed study interventions**	**3.3 Treating early-BCRL with intensive-DLT - is it worth it?**
W10	Thought it was worth considering, if past radiotherapy and chemotherapy	Time commitment and arm bandaged for weeks.<br>Didn't comment on modified DLT.	Would be worth having DLT if arm was not swollen after treatment. But aware DLT does not guarantee resolution of swelling and was not sure she'd get sufficient benefit for the effort - didn't think arm was problematic enough to justify DLT
W11	Acceptable as commitment is similar to radiotherapy which had already been achieved	Having bandaging, because of difficulties with clothes and having a shower. More acceptable to have pump as used to daily travel for DXT. "*Because it's more akin to radiotherapy: you're just travelling in every day. You've done it; you've already been there."*<br>Felt this was at least a treatment, rather than none (i.e. self-care). "*It just seems to be <u>a</u> treatment as well, as opposed to none."*	Only worth having DLT if swelling causes enough discomfort.<br>*"I think the bandaging looks positively medieval and not 21st century medical science. So, the thought of having to do that if you aren't in enough discomfort really puts you off. If it was offered to me, no."*

Theme 3 (cont): Acceptability of proposed study (FGW2)			
**Case name**	**3.1 Acceptability of participation in future lymphoedema research**	**3.2 Burden of proposed study interventions**	**3.3 Treating early-BCRL with intensive-DLT - is it worth it?**
W12	Felt timing might not be right if recently finished cancer treatment, as need to focus on getting back to normal.	Main problems: time commitment and inconvenience of bandages worn all day. Weekends were difficult. Bandages affected mobility and function, with difficulty getting dressed and finding clothes to fit.<br>Modified DLT was an easy, very relaxing process, that was not intrusive, and helped effectively address her symptoms. She did not find MLD and pump daily to be onerous, other than travel, so thought this a much more acceptable study intervention."<br>*"I would wonder whether the pump one would not be an easier treatment to accept at that point, perhaps a daily pump, because it's less intrusive.(…) It's also quite relaxing. When you have your arm in the sleeve and it's pumping away – I have fallen asleep. So, it isn't onerous, apart from the travelling. It's actually very, very easy. So, that I think makes life more acceptable."*	Had experienced several courses of phase 1 DLT and modified DLT, and felt these were at least doing something. However, was unsure whether she would have accepted early DLT, despite offering greater improvements than self-treatment and reassurance she'd tried everything possible.<br>Would definitely have accepted early DLT if she'd known the problems that would develop later that might be prevented by early DLT.<br>*"It would be an interesting one to know if I had had (intensive-DLT) as standard treatment at the very beginning of the first diagnosis whether I would have developed it more seriously later. (…) When I've had the bigger interventions, as it were, I felt at least I'm doing something."*
W13	Thinks practitioners suggesting the trial to women would indicate practitioners believe the study intervention was better than usual care.	Difficult if still working. Would be hard to manage with bandages if live alone. Modified DLT not discussed.<br>*"To be honest with you, I would certainly have (had intensive-DLT) if I wasn't able to type anymore, because that is so fundamental to my working life. But with my symptoms as they are, no, unless two weeks of it would guarantee that I wouldn't have any more symptoms ever again, then that would be a small price to pay"*	Would have accepted early DLT if unable to type or continue working due to lymphoedema symptoms or if treatment would improve swelling.<br>Now that symptoms are stable, would only be willing to have DLT if it would resolve symptoms with no need for ongoing hosiery; in which case, she would be eager to have DLT.

Theme 4 (women's individual responses): Living with lymphoedema (FGW1)				
**Case name**	**4.1 Physical and psychological consequences of BCRL**	**4.2 Learning to live with lymphoedema**	**4.3 Hopes, expectations and beliefs about lymphoedema treatment**	**4.4 Impact of knowledge deficit**
W1	Range of symptoms: arm swollen, heavy, tiring & painful especially when lifting things, pins & needles in hand; also has breast oedema. Had to stop wearing rings for a while. Hands weak so easily drops things and couldn't lift 2-yr old son.<br>Fluctuating hand swelling. Increased swelling with activity. Chores impact BCRL. Difficulty with household chores. Limits activities and has to ask for help.	*"But I came to terms with (lymphoedema) like, 'Okay I'm alive. I can live with wearing sleeves'…I make do with what I have and be grateful."*<br>*"(BCRL) worries me, but not as much as thinking: will the cancer be back."*	At the start my hope *"was very high, that I was going to be cured."*	Need explanation of lymphoedema and treatment options, including how to treat breast oedema and info re treatment outcomes
W2	Swelling in dominant arm so hard to do chores. Can't lift shopping or carry things with that arm. Needs help with chores and hesitant about what can do without impacting BCRL.<br>Has pins & needles in hand, rings are tight. Can't wear ¾-length or long sleeves, even in winter, as arm big & thick and clothes don't fit. So lives in short sleeves & cardigan.	Daughter helps with ironing and other chores, particularly lifting. Husband and daughter had cancer at the same time as me, so LO not a priority.	*"I don't know what mine are"* (treatment goals).<br>Believe it is important to follow HCP guidelines.	I didn't know about the LSN. I know another hospital which had no information about risk of lymphoedema [no problems with hosiery – has good chemist]
W3	Swollen hand is weaker & has pins & needles. Easily drop things and hard to do chores. Can't wear rings. Need clothes with big sleeves to fit over arm and hosiery.	*"Like you (W2), ironing's a bit of a whatsit, but I've got no one else to do it so I have to do it anyway. But other than that, I live with it"*	Told lymphoedema was lifelong. Agreed with researcher summary that patient focus is on getting treatment rather than having goals for treatment	HCP didn't recognise cause of oedema. Wants more information about treatment. Is difficult getting hosiery.

Theme 4 (cont): Living with lymphoedema (FGW1)				
**Case name**	**4.1 Physical and psychological consequences of BCRL**	**4.2 Learning to live with lymphoedema**	**4.3 Hopes, expectations and beliefs about lymphoedema treatment**	**4.4 Impact of knowledge deficit**
W4	Difficulty controlling swelling.<br>Fear of BCRL getting worse if lift heavy things so need help from husband and children with chores and lifting.	"I don't ever forget I have lymphoedema, but I'm alive."<br>Difficult knowing what causes what symptom changes<br>Wary of impact of chores on lymphoedema, so asks for help.<br>*"We're aware of what might happen and therefore we let other people do these things. We are naturally hesitant about doing the ironing or whatever."*	Thought swelling would just go away as it did the first time following treatment. Questioned whether prompt treatment would have had a similar effect in second episode<br>Don't know what to expect now from treatment. Trusts practitioners to know what is best<br>Education of GP would be helpful and also reduce pressure on HCP resources.	Wary of doing chores for fear of impact on swelling if do heavy chores. Asks others to do chores. People lack info about long-term implications of lymphoedema and what action to take if problems arise.
W5	Swollen arm with internal ache & pain which gets quite intense by end of day.<br>Have been through so many things that lymphoedema is just another thing to deal with.<br>*"I had psychological problems as well, being that I was very worried about it, very depressed. I thought it was curable and I'd never heard of it. Later I didn't know what to expect."*	Has learned to live with it over past ten years.<br>Joined the LSN to gather information.<br>Uses chores to improve treatment effect.<br>*"I manage to do most things. Ironing I don't particularly like, but I do it because I believe that any form of housework, as long as it's not really excessive, helps."*	Initially thought it was curable. Found treatment a little trial and error. Now believes swelling is at an acceptable level and can't see another way forward.<br>Believes women want to feel they have done all they can to treat their lymphoedema. Also believes exercise and household chores help to treat lymphoedema.	It took a long time to find out about lymphoedema treatment and treatment decision-making.<br>It is vital to know why treatments are needed and what can be expected from treatment.

Theme 4 (cont): Living with lymphoedema (FGW2)				
**Case name**	**4.1 Physical and psychological consequences of BCRL**	**4.2 Learning to live with lymphoedema**	**4.3 Hopes, expectations and beliefs about lymphoedema treatment**	**4.4 Impact of knowledge deficit**
W6	BCRL has no impact other than having to wear sleeve	Has not altered lifestyle or stopped things because of lymphoedema. Considers main problem to be weight increase, not lymphoedema. *"Because of my lifestyle I've never stopped doing whatever I did before. I've never had time to think about it. (…) I've had the babies all the time. I've got a couple of big dogs. And I've just carried on as I always did. I've never changed anything."*	Did not want to know about lymphoedema. Thought herself lucky not to need bandaging.	Put on BP meds by GP then told by LO clinic that the tablets were bad for LO
W7	*"The psychological impact is powerful. It changes your femininity."* Thinks stress may affect my swelling & make it worse. *"It's all part of the whole deal, isn't it, with breast cancer: it challenges your femininity; and then you end up with this huge arm. People used to say to me, "_, your arms are so slim it doesn't matter". And I'd say, "I accept that. But actually the impact of lymphoedema is you've got a whole load of lymph here causing an arm that's bigger than the other one, and it shouldn't be there."* The look of hosiery is important, but comfort comes first. Hosiery is visible and people ask questions.	[n.d.]	Had no understanding of lymphoedema. Dreams of having enough money to get 3 weeks lymphoedema treatment in Austria.	Information can get lost or overlooked, and sometimes too much to take it all in. Didn't want to know statistics about lymphoedema.

Theme 4 (cont): Living with lymphoedema (FGW2)				
**Case name**	**4.1 Physical and psychological consequences of BCRL**	**4.2 Learning to live with lymphoedema**	**4.3 Hopes, expectations and beliefs about lymphoedema treatment**	**4.4 Impact of knowledge deficit**
W8	I think it would be valuable to monitor body image every 6-12 months. The questions should focus on how you feel re swelling in your body…	*"I feel like I'm being a good woman at the moment; (the sleeve is) on every ruddy day. But at some point I suspect I'll slip away."*	No expectations expressed.	Need information about what is treatment. Knowledge about how to self-monitor changes to swelling would improve understand of what treatment changes needed
W9	It's a chronic condition, for life. I need to wear the dreaded sleeve.<br>*"To me the really important one, I always refer to it as my visiting card for cancer, is the daily reminder that I've had it. It is really difficult to move on when you've got this daily reminder."*	Found helpful information at LSN.<br>Having to battle with other cancer side-effects, so lymphoedema almost a side issue.	Knew it was a chronic condition, for life.<br>Believes lymphoedema can improve and that it is definitely controllable.	Received mixed messages about importance of skin care and exercise.<br>*"the first question she asked, "Have you been carrying a lot of stuff?" And I said, "Yeah, I've been carrying all these pots and gardening and everything". And she said, "Well, that's it then, isn't it?" ((Laughs)) So, the advice has improved now I think."*
W10	Upset with lymphoedema and lack of progress. Can't have arm massaged. BCRL affects my chronic fatigue. Almost scared to use arm and careful about what I carry on arm.<br>It's a reminder of breast cancer and causes difficult conversations.	Upset with lymphoedema and lack of progress.<br>*"Do you find that you have to limit the use of your arm for picking up weights? (…) I'm always careful of my arm on weight. I use my left arm all the time for weight. I'm almost scared to use it."*	Scared to use arm because of impact of chores on lymphoedema.<br>Believe lymphoedema can be controlled and that treatment is just to control swelling.	Not given treatment immediately as HCP thought swelling might spontaneously resolve. Is unclear why swelling happens and whether arm can be massaged. Told she can only have 2 sleeves annually.

Theme 4 (cont): Living with lymphoedema (FGW2)				
**Case name**	**4.1 Physical and psychological consequences of BCRL**	**4.2 Learning to live with lymphoedema**	**4.3 Hopes, expectations and beliefs about lymphoedema treatment**	**4.4 Impact of knowledge deficit**
W11	"It's almost separate to your quality of life, because your quality of life can be quite good but you body image perception could still be quite low even at the same time."	[n.d.]	Believes she's been given maintenance treatment from the start and not real treatment	Frustrated by lack of information about other treatment options and conflicting advice re lymphoedema. Disappointed that she knows more than HCP seem to. *"I am just frustrated with the amount of conflicting advice you get from every single person you see along the process.* (*…group discussion…*) *They don't assess that you don't need to be talked to like an 8-year old. I have read publications (…) Basically I was disappointed because I felt I knew more than she (the lymphoedema practitioner) did. But that doesn't mean she wasn't the loveliest person who mopped up my tears."*
W12	Arm swells more in the heat. Had frequent cellulitis & on antibiotics for about 3 years – now controlled & no infection for a while. Arm aches and swells if carry things/weight. *"It's difficult when people ask about my arm. 'This is a little residue' and as I said quite a complicated thing to explain to relative strangers."* Swelling worsened after cancer recurrence.	LO treatment may not be the priority when trying to get life back to normal after main cancer treatment. Uses exercise to keep arm flexible, while not doing too much.	Believes slight swelling can be maintained and, if it worsens, can get better. Bigger interventions felt like something was being done, whereas sleeve doesn't always feel it is enough.	Agreed with W9 about starting to get to know and understand body. *"I think there's a difference though, isn't there, between carrying weights and more general exercising."*

Theme 4 (cont): Living with lymphoedema (FGW2)				
**Case name**	**4.1 Physical and psychological consequences of BCRL**	**4.2 Learning to live with lymphoedema**	**4.3 Hopes, expectations and beliefs about lymphoedema treatment**	**4.4 Impact of knowledge deficit**
W13	Hand aches. Don't knit any more as get cramped up.<br>Wear compression gloves so I can keep typing – my biggest problem is finding way to keep typing.<br>Depressed by arm swelling. Think stress & depression cause increased swelling.<br>Frustrated that BCRL developed when told had very low risk.<br>Lymphoedema is reminder of cancer and that not fully recovered:<br>*"Lymphoedema gives you a sense that you haven't recovered and you won't recover."*<br>Dislikes hosiery. *"(hosiery) just is the colour of illness…nobody ever wears anything that colour unless there's something wrong with them."*	*"I find the advice on weight really confusing and difficult. (…) I have been very diligent about the exercising. But I'm beginning to wonder whether (…) I ought to be using my right hand more and I'm overprotecting it."*	Lymphoedema is chronic but want hope, not a bleak diagnosis. Nobody actually knows what outcomes are possible.<br>Self-care was not presented as treatment, altho she views them that way.<br>*"I'm feeling quite annoyed actually that there is such a bleak prognosis given – that it's only going to get worse and worse and that's it. And actually I would rather (be told): 'the truth is we don't really know'. ((Laughs)) I would find that (a) more hopeful, and (b) more truthful. It seems to me those are two very important things. (…) As human beings we don't just want the caution; we want, we need a bit of hope." ((Laughs))*	Felt overwhelmed by the amount of information given, and not easy to understand and apply to life. Wants more written information to reduce confusion.<br>Unsure of impact of actions on lymphoedema and if overprotecting arm.<br>*"I find the advice on (lifting) weight really confusing and difficult. (…) It is quite hard to apply and understand and work out what to do." (…) "How heavy is heavy?" ((Laughs))*

(b) Matrices for practitioners, focus group 1 (FGP1), focus group 2 (FGP2) and focus group 3 (FGP3)

Theme 1: Usual care offered to women with BCRL					
**Case, group**	**1.1 'Usual' lymphoedema treatment**	**1.2 Variations to 'usual' BCRL treatment**	**1.3 Women's adherence to recommended treatment**	**1.4 When is intensive-DLT offered?**	**1.5 What does the term 'treatment' mean?**
**Focus group 1** (FGP1)[1]	Self-treatment. Hosiery is standard, although may be omitted if swelling negligible. Some consider exercise to be part of usual care, others add it later. May also include SLD, KT, although not necessarily at first appointment – will depend on clinical presentation.	Variations to treatment focus on self-care, mainly by adjusting hosiery or adding SLD. Different types and compression classes tried.	3/4 practitioners group discussion of reasons for non-adherence: burden of wearing hosiery, lack of knowledge/understanding. Group agreed it was important to assess adherence, understand/ identify reasons for non-adherence, particularly before offering DLT in case they don't comply with hosiery post DLT.	Practitioners put patients on wait list for treatment when they meet prespecified criteria, including size (typically ≥20%EAV), fibrosis, shape distortion, whether adherent to wearing hosiery.<br>Once on wait list, will be prioritised by severity or urgency, and may consider personal circumstances.<br>Rarely offered, and not as initial treatment.	Practitioners generally referred to patient input only by naming individual treatment components. PN1 used 'treatment' for both practitioner and patient input.
[1]FGP1 included 4 lymphoedema practitioners with nurse backgrounds from 3 hospital-based and 1 hospice/community-based services. Three treat only patients with cancer-related lymphoedema and one treats all types and causes of lymphoedema. All services provided comprehensive treatments, including intensive-DLT, although one practitioner only provides phase-2 DLT, the maintenance phase.					

Theme 1: Usual care offered to women with BCRL					
**Case, group**	**1.1 'Usual' lymphoedema treatment**	**1.2 Variations to 'usual' BCRL treatment**	**1.3 Women's adherence to recommended treatment**	**1.4 When is intensive-DLT offered?**	**1.5 What does the term 'treatment' mean?**
**Focus group 2** (FGP2)[2]	Group agreed usual treatment is self-management with hosiery, exercise, skin care ± SLD + education 1-1 or as group. Emphasis on assessment prior to treatment so can individualise care. Treatment focuses on self-management. Mostly use round knit hosiery. DLT not part of usual care.	Custom garments used when no suitable readymade garment available. Patch test and consider kinesiotape. Myofascial release used by 3 physios. No mention of intensive-DLT as treatment variation, although it is a treatment provided by each clinic.	Women may try to disguise non-adherence but practitioners recognise it and the consequences.	Offered to patients who meet prespecified criteria, including size (typically ≥20%EAV or >30%EAV), fibrosis, shape distortion, whether adherent to wearing hosiery. One practitioner also offers intensive-DLT when symptoms progress with self-treatment; unclear whether other services also do this. Practitioners use wait list to manage limited resources, with intensive-DLT rarely offered, and not as initial treatment. One practitioner modifies intensive-DLT to include SLD or IPCT when lack resources to provide MLD.	No group discussion about treatment terminology, but throughout the discussion the term 'treatment' referred to practitioner input and a variety of terms such as 'management' or 'self-management' referred to patient input.

[2] FGP2 included 4 practitioners with physiotherapy backgrounds and one nurse who all provided comprehensive treatment, including intensive-DLT; two also provided private lymphoedema treatment. They represent 3 hospital-based, 1 hospice and 1 Any Qualified Provider service, with three offering treatment to all types of lymphoedema and two limited to cancer-related lymphoedema. Four had >5 years' experience, and 1 had 2-5 years' experience.

Theme 1: Usual care offered to women with BCRL					
**Case, group**	**1.1 ‘Usual’ lymphoedema treatment**	**1.2 Variations to ‘usual’ BCRL treatment**	**1.3 Women’s adherence to recommended treatment**	**1.4 When is intensive-DLT offered?**	**1.5 What does the term ‘treatment’ mean?**
**Focus group 3** (FGP3)[3]	Mixed views on whether to use hosiery as initial treatment and if hosiery+skin care was sufficient to improve mild/minimal lymphoedema.<br>They individualise treatment according to clinical presentation and patient needs. Education lymphoedema and self-management is a key component of treatment.	Consider whether need intensive-DLT or components of it, e.g. MLD.<br>May add or alter hosiery prescription to achieve a reduction in arm volume and to improve adherence. May teach SLD if not taught at 1st appointment.	No group discussion although individual practitioners noted the importance of addressing it with education or adjusting treatment. Non-adherence would affect likelihood of receiving intensive-DLT	Typically patients are added to wait list when size ≥20%EAV, fibrosis, shape distortion. Some practitioners consider texture/density more important indicator for treatment than %EAV.<br>Practitioners hope they can improve lymphoedema with self-treatment, manipulating compression garments, so intensive-DLT is not required.	Group discussed terminology and purpose of patient input. Regardless of terminology, they agreed their expectation was that patient self-treatment with hosiery would reduce oedema and soften tissues, i.e. decongestion.<br>Practitioner input was always referred to as ‘treatment.’ Some practitioners referred to patient input as ‘treatment’ and others used ‘management’ or ‘care’.

[3] FGP3 included 4 practitioners with nurse backgrounds and >5 years’ experience. They all provided comprehensive treatment, including intensive-DLT. Two offered treatment of all lymphoedema and 2 were limited to cancer-related lymphoedema. They represent 2 hospital, 1 hospice/community and 1 community-based services.

Theme 2 (practitioner): Monitoring outcomes				
**Case, group**	**2.1 Assessment methods used**	**2.2 Challenges for assessment/monitoring**	**2.3 Expectations of treatment**	**2.4 Priority outcomes**
FGP1	Group measures arm size and monitors fibrosis and patient perspective. No mention of specific QOL tools used.	No group discussion.	2.3.1. self-treatment: achieve stable swelling.<br>2.3.2. intensive-DLT: mixed views regarding expected outcome, including hope for reduction, but mostly expect it will achieve stability of BCRL symptoms (prevent them worsening) and improve quality of life.	Group listed a range of assessment options although did not clearly indicate which were important outcomes or identify what improvement of symptoms looks like.<br>Group agreed it was important to objectively monitor arm volume and changes to arm shape and tissue texture (fibrosis), and monitor the patient perspective. Beyond using QOL tool, the group had no experience of using patient reported outcome measures (PROMs).
FGP2	No group discussion.<br>All practitioners performed clinical assessments, typically measuring arm volume, tissue tone and QOL. They use their findings to determine what treatment to offer.	No group discussion. Individuals unsure of appropriate outcomes for determining treatment effectiveness	No group discussion of expectations for general treatment or intensive-DLT.<br>No group discussion about achieving decongestion of the arm or defining a clinically significant outcome.	Practitioners talked about a range of tools, although unclear whether these were current tools or things methods that had tried.<br>Not explored in discussion.

Theme 2 (practitioner): Monitoring outcomes				
**Case, group**	**2.1 Assessment methods used**	**2.2 Challenges for assessment/monitoring**	**2.3 Expectations of treatment**	**2.4 Priority outcomes**
FGP3	No group discussion, but frequent mention of measuring arm volume. Practitioners did not each list all the assessment methods they used but instead mentioned methods not already listed.	No group discussion, just individual statements about issues with different assessment methods, including difficulty identifying volume of minimal swelling, interpreting BIS results, the usefulness of MYMOP if women all report different issues.	Group struggled to quantify expected treatment outcomes.<br>2.3.1. self-treatment: group generally agreed that, at minimum, self-care treatment with hosiery should control swelling and prevent deterioration. Where women used stiffer/thicker garments, they expected oedema to reduce and fibrosis to soften.<br>2.3.2. intensive-DLT: a clinically significant outcome for intensive-DLT was reduction in swelling to minimal/no oedema, although group unsure if this was realistic.	No group discussion, but general agreement about importance of measuring arm volume. Other outcomes to consider included time to discharge from clinic, pitting oedema, fibrosis.

Theme 3 (practitioner): Acceptability of proposed study			
**Case, group**	**3.1 Acceptability of proposed lymphoedema trial**	**3.2 Burden of proposed study interventions**	**3.3 Potential benefit of initial intensive-DLT**
FGP1	Group recognised benefit of research to increase knowledge.<br>They thought patients were likely to want to participate in study but were concerned about capacity of clinics to provide study intervention without disadvantaging patients already waiting for intensive-DLT.	Time commitment and impact of bandages on daily life.<br>Group were concerned patients might not be ready for early intensive-DLT and that daily treatment may act as reminder of cancer. Practitioners would need to monitor bandages to ensure correct pressures.<br>Practitioners felt modified intensive-DLT may be more acceptable to patients, but they had little experience of using IPCT and did not know the evidence base for it.	Group considered early intensive-DLT could be worthwhile to prevent future problems from tissue changes (e.g. fibrosis), but questioned whether it was necessary and appropriate as 1st treatment. They thought women newly presenting for treatment would find early intensive-DLT acceptable.
FGP2	Considered patients are likely to want to participate and gain additional treatment.<br>Practitioners think patients are used to trials, so should be given option to participate in or reject study.<br>More likely to get recruits for modified intensive-DLT.<br>Group had lots of questions about how different elements of study interventions would be standardised.	Travel and time commitment of frequent appointments could be challenging. Bandaging affects work and daily life.<br>No group discussion of modified intensive-DLT, although practitioners felt modified intensive-DLT would be more acceptable to patients than compression bandaging.	Uncertain of acceptability of intensive-DLT for patient. Group had mixed views, and questioned whether early intensive-DLT was appropriate, e.g. would early intensive-DLT prevent future problems? Is early intensive-DLT necessary when self-treatment can achieve a good outcome? Is early intensive-DLT ethical when others patients are already on waiting list for intensive-DLT?

Theme 3 (practitioner): Acceptability of proposed study			
**Case, group**	**3.1 Acceptability of proposed lymphoedema trial**	**3.2 Burden of proposed study interventions**	**3.3 Potential benefit of initial intensive-DLT**
FGP3	Group agreed with proposed eligibility criteria. They highlighted challenges for standardising components of the study intervention. They were not sure whether early intensive-DLT would be acceptable to practitioners who already operate waiting lists for intensive-DLT.	Not sure whether intensive-DLT would be acceptable to patients, due to time commitment and effect on daily activities.<br>Practitioners lacked knowledge and experience of IPCT as rarely used.	General agreement that early intensive-DLT would prevent future problems, including fibrosis.<br>Unsure of benefit/value of early intensive-DLT for women with mild or minimal BCRL.

Theme 4 (practitioner): Lymphoedema service issues				
**Case, group**	**4.1 Characteristics of women newly presenting for BCRL treatment**	**4.2 Changing nature of lymphoedema practice**	**4.3 Challenges and frustrations of managing limited resources**	**4.4 Patient education**
FGP1	No group discussion. Individuals mentioned that most women present early with mild swelling, although some may have mild size but firmer more severe symptoms.	Not discussed	No group discussion. Services have strategies to manage limited resources, including wait lists for intensive-DLT, use of 2x/wk Coban2 bandaging instead of 5x/wk short-stretch bandaging, patient asked to obtain hosiery via GP prescription to reduce lymphoedema clinic costs.	No group discussion. Individual comments support the importance of patient education. Mixed views regarding amount and pacing of information to be given to patients.<br>Limited group discussion about mistaken patient views at 1st appointment regarding treatment.
FGP2	No group discussion. Some reported that women generally seen early with mild symptoms.	No group discussion. Two practitioners stated patients now present earlier with milder symptoms	Main challenge is requirement to obtain hosiery via GP, to reduce treatment costs for clinic	Group agreed on importance of education regarding what is lymphoedema and self-management
FGP3	Mild symptoms, typically with soft oedema and <10% EAV. Many presenting with breast oedema as main problem ±arm swelling	Patients now present earlier and with very mild arm swelling; few now present with severe oedema. Easier to treat the milder swelling. Increased numbers presenting with breast oedema as main or only symptom.	Practitioners frustrated that lack of resources prevent them offering early intensive-DLT.<br>To manage resources, they have implemented discharge policies and community hosiery prescription.	Practitioners identified various tools used, including LSN and Macmillan leaflets, and many developed their own leaflets. They clearly valued education.

Theme 1: (practitioner's individual responses): Usual care offered to women with BCRL (FGP1)					
**Case ID**[1]	**1.1 'Usual' lymphoedema treatment**	**1.2 Variations to 'usual' BCRL treatment**	**1.3 Women's adherence to recommended treatment**	**1.4 When is intensive-DLT offered?**	**1.5 What does the term 'treatment' mean?**
PN1	Self-treatment with sleeve ±glove (omitted if no visible/palpable oedema), BCRL exercise regime (no detail), education to increase patient engagement ± SLD. Initial treatment includes infection prevention advice, armsleeve±glove, skin care, exercise ±SLD.	Uses all treatments except LLLT and IPCT. May add kinesio-tape or teach SLD if woman wants. Intensive-DLT is MLLB, MLD, daily for 2-3 weeks. Has 3 wait lists for (1) MLD +bandaging, (2) bandaging only, (3) MLD only.	Focuses on education to encourage adherence and empowerment for self-management. Assumes non-compliance (sic) is likely reason for poor treatment progress so first will check reason for not wearing garments, e.g. psychological issues, burden of wearing sleeve.	Pre-specified criteria used to decide whether to offer intensive-DLT and to prioritise wait list/urgency of treatment. Will consider patient circumstances. *"They will have to meet certain criteria (for treatment). For example, over 20% swelling, requires hospitalisation, shape distortion, fibrotic tissue. So if they meet eight out of fifteen criteria, then they will go forward for treatment. And they will be classified also as less urgent, urgent or ASAP"*	Uses 'treatment' and 'management' terms interchangeably to describe input by both practitioner and patient
PN2	Self-care: sleeve ±glove using different hosiery fabrics, KT, SLD; only teaches SLD if patient keen to learn. Focus on encouraging independence. Initial treatment is sleeve±glove.	Consider alternative hosiery type, add kinesio-tape, SLD, or whether intensive-DLT is required. Intensive-DLT includes Coban bandaging 2-3x/wk.	If poor progress, will check compliance (sic) & hosiery tolerance, what they can/will wear; also check adherence to hosiery before offering intensive-DLT. Thinks women don't want garments.	Decision to offer intensive-DLT is based on degree of swelling, when hosiery cannot be fitted or does not control symptoms, but no set policy. Also considers likely garment adherence post-intervention and whether psychologically able to cope with intensive-DLT. *"We need to be convinced that the patient will be able to tolerate the garments and that they will wear it, before we would think about going to the next stage of offering DLT if they needed it… before we invest all that time and effort into them"*	Used 'treatment' to refer only to practitioner input. One mention of 'self-care;' no generic term for describing patient input

Theme 1: (practitioner's individual responses): Usual care offered to women with BCRL (FGP1)					
**Case ID**	**1.1 'Usual' lymphoedema treatment**	**1.2 Variations to 'usual' BCRL treatment**	**1.3 Women's adherence to recommended treatment**	**1.4 When is intensive-DLT offered?**	**1.5 What does the term 'treatment' mean?**
PN3	Self-management with sleeve (may omit if symptoms mild), SLD, education.<br>Initial treatment is education, sleeve (if arm swelling), patch test kinesio-taping for hand/finger swelling	Offers all treatments except LLLT & IPCT. Varies garment prescription; may start with light hosiery and increase to the required garment or postpone fitting sleeve to 2nd appointment.<br>SLD added at 6-week review if required, kinesio-tape for hand/finger swelling.<br>Intensive-DLT customised by varying treatment course duration and bandaging frequency according to patient needs, e.g. personal circumstances bandage tolerance, mobility. Does not use Coban2.	Adopts a gentle approach tailored to patient capacity to promote 'concordance' (sic). *"I do the gentle-gentle, because in the long term I seem to find that patients come on board more."*<br>Thinks non-compliance (sic) with hosiery and not understanding their BCRL treatment are the most likely reasons for poor progress. So assesses adherence to prescribed hosiery before offering intensive-DLT.	Criteria for intensive-DLT include severity of swelling, shape, EAV%, mobility, function and whether likely to wear hosiery post treatment.<br>Will also consider psychological readiness, availability of help at home, and priority of other health issues.<br>Will also consider intensive-DLT if hosiery distorting arm shape.	Used 'treatment' only to refer to practitioner input.<br>Considers ideal treatment to be daily intensive-DLT
PN4	Self-management with armsleeve, exercise (no details), education re treatment and rationale. Gives women prescription to get hosiery on FP10.<br>Initial treatment is a prescription for armsleeve via GP.	Alters hosiery prescription if not improved within 3 months. May add exercise or MLD before considering intensive-DLT. May use soft bandaging to reduce risk of lymphorrhoea if skin is fragile or broken.<br>Intensive-DLT includes Coban2 bandaging 2x/wk ±MLD. Works at multiple sites so unable to bandage daily.	Depending on psychological state of patient, may need to repeat information or give more until they can take it in.	Criteria for intensive-DLT: >20%EAV, distorted shape, fibrosis. May also consider treatment if skin tense or friable.	'Treatment' used once to describe patient input.<br>Considers ideal treatment to be bandaging and MLD

Theme 1: (practitioner's individual responses): Usual care offered to women with BCRL (FGP2)					
**Case ID**	**1.1 'Usual' lymphoedema treatment**	**1.2 Variations to 'usual' BCRL treatment**	**1.3 Women's adherence to recommended treatment**	**1.4 When is intensive-DLT offered?**	**1.5 What does the term 'treatment' mean?**
PP1	Self-management: skin care, exercise (i.e. exercise they enjoy), compression hosiery, education with self-management advice;<br>Typically provides round-knit garments for early/mild BCRL. Provides prescription details to get 2nd garment via GP/local pharmacy.<br>Initial treatment includes hosiery, education ± kinesio-tape patch test. Rarely teaches SLD at 1st visit.	Use firm round-knit garment before trying custom-made. May add KT, although not under hosiery.<br>Intensive-DLT is daily treatment for 3 weeks with comprilan bandaging±MLD, exercise; if use Coban bandaging then twice weekly treatment. Don't always need full 3-wk treatment.	[n.d.]	Sometimes have resources only for bandaging so will include SLD or IPCT instead of MLD.	'treatment' used only to refer to practitioner input, whereas patient input referred to as '*standard* treatment', 'self-care', 'self-management, and 'self-maintenance'.<br>Thinks current approach gives optimal treatment for mild BCRL, although, in ideal world, would offer DLT/MLD without wait lists as needed for those with severe symptoms.
PP2	Self-management: hosiery, skin care, education, specific and general exercise, teach SLD, healthy eating + weight management. Informed about support group. Signposting to support group and Macmillan. Use of written information. Annual review.<br>Initial treatment is individualised self-management, as above.	Vary hosiery prescription, consider ACWD or kinesiotaping. Offer myofascial release treatment if tight.<br>Has begun transfer to FP10 garments, where possible, for women to get from GP.<br>Intensive-DLT is 2-3x/week bandaging±MLD. Will add IPCT+exercise+KT if can't provide MLD.	Can usually tell when patient is not using hosiery. Will ask patient to keep a diary log of exercise during DLT, to promote adherence.<br>Thinks it would be useful to research how invested women are in self-management.	Criteria for intensive-DLT is >30%EAV, with priority to treat fibrosis, pain, discomfort.<br>Otherwise, only offer DLT if have capacity.	Referred to patient input as self-management.<br>*"We do lots of self-management and some intensive; although just because of time we have to limit it at the moment to those who have more severe problems."*

Theme 1: (practitioner's individual responses): Usual care offered to women with BCRL (FGP2)					
**Case ID**	**1.1 'Usual' lymphoedema treatment**	**1.2 Variations to 'usual' BCRL treatment**	**1.3 Women's adherence to recommended treatment**	**1.4 When is intensive-DLT offered?**	**1.5 What does the term 'treatment' mean?**
PP3	Self-treatment with hosiery (mainly circular knit), education, skin care, general and ROM exercise. May teach SLD at later appt<br>First appointment focused on assessment and teaching about lymphoedema and self-care, so only fit hosiery if time available. Follow-up at 6-9m intervals until stable, then annually	Adjust hosiery prescription; use mainly round-knit but also flat-knit garments, with no restrictions to ordering hosiery. Also offer myofascial release & scar management<br>Intensive-DLT is 3-wks daily short-stretch bandaging, MLD, exercise, skin care. Teach SLD in final week of treatment.	*"We are really, really into explaining that it's very important to self-manage, and that they understand that we are here to support them but they actually need to do their part, otherwise it's very difficult to maintain."*	Criteria for intensive-DLT: >20%EAV, distorted shape, fibrosis	'Treatment' used only to refer to practitioner input, such as intensive treatment.<br>Patient input referred to as 'self-care management' or 'self-management'.<br>*"We offer here intensive treatment and self-care management"*
PP4	Self-treatment with hosiery (omitted if 'light' oedema), SLD, lymphoedema-specific exercise (to aid drainage), nutrition advice, education about self-management.<br>Initial treatment is education about lymphoedema and self-care, and teach SLD.<br>May use hosiery with GP prescription.	Varies hosiery prescription; uses round knit garments to get patient used to hosiery, change to flat-knit if worsen and post-intensive-DLT. Can individualise garments using sewing machine. May add ACWD or teach patients to self-bandage. Offers myofascial release if tight.	*"There's no point bandaging someone if they're not going to exercise with it".*<br>Many patients do not regularly wear hosiery or attend follow-up. They need to reach point where prepared to self-manage lymphoedema.<br>Believes patients more likely to be adherent with hosiery if start with round knit rather than flat-knit.	Criteria for intensive-DLT include fibrosed tissues or progression of BCRL with self-management.	'treatment' referred only to practitioner input whereas 'management' or 'self-management' referred to patient input<br>Ideal treatment would include MLD for everyone

Theme 1: (practitioner's individual responses): Usual care offered to women with BCRL (FGP2)

Case ID	1.1 'Usual' lymphoedema treatment	1.2 Variations to 'usual' BCRL treatment	1.3 Women's adherence to recommended treatment	1.4 When is intensive-DLT offered?	1.5 What does the term 'treatment' mean?
PN5	Self-treatment with hosiery (many flat-knit, 2 garments each time), skin care, exercise, position & movement, SLD taught at next appointment.<br>Initial treatment follows a formalised treatment pathway which includes education. All patients invited to 1.5hr workshop addressing all self-care issues, with practice time for SLD and 30 min Tripudio exercise class.<br>SLD not taught at first appointment	Adjust hosiery prescription, add ACWD, discuss intensive-DLT if needed. Uses mainly flat knit hosiery, with made-to-measure garments obtained if not suitable ready-made garment. Mostly obtains garments via GP prescription as clinic hosiery budget is very small.<br>Intensive-DLT is 3 wks bandaging+MLD ±kinesio-taping ±Laser therapy, with the 3rd week used to transition to custom-fit sleeve. Treatment components and duration will depend on individual; will consider impact on employment. Usually bandage with comprilan, very occasionally Coban.	[n.d.]	Criteria for intensive-DLT based on "5 'S' score" to determine lymphoedema severity. Treatment approach is based on LF (2006) consensus document	'treatment' used to refer to DLT and practitioner input, whereas patient input referred to as 'self-focus'.<br>Ideal treatment would be intensive-DLT for all with severe swelling, plus maybe kinesio and laser therapy, whereas those with mild-moderate BCRL would have self-treatment with hosiery, skin care, SLD, exercise.

Theme 1: (practitioner's individual responses): Usual care offered to women with BCRL (FGP3)

Case ID	1.1 'Usual' lymphoedema treatment	1.2 Variations to 'usual' BCRL treatment	1.3 Women's adherence to recommended treatment	1.4 When is intensive-DLT offered?	1.5 What does the term 'treatment' mean?
PN6	Self-care with hosiery (round or flat knit according to clinical presentation), skin care as priority, and exercise (general and lymphoedema-specific). Flat knit used if arm soft and fatty, minimal swelling (<10% EAV) with fibrosis. Initial treatment is exercise, skin care and compression sleeve if required	Change prescription for hosiery. May add ACWD, teach self-bandaging, ± MLD or teach SLD instead. "*My decision on what type of sleeve is mainly due to density of tissues and capacity of the patient.*" Intensive-DLT is MLD, short-stretch compression bandaging, skin care, lymphoedema-specific exercise.	[n.d.]	Criteria for intensive-DLT: skin and tissue texture/density, distorted shape, with less focus on %EAV.	Thinks meanings have changed over years to indicate treatment component, e.g. 'maintenance phase' means self-treatment phase. "*Perhaps we're still using the old terms but we're not really meaning it in the way that it used to be. But it's a language that we sort of all understand. ((All agree)) If you're talking about the maintenance phase you're not going to be talking about an intensive course of bandaging and MLD. (…) So the term you are using is much better: the self-care phase.*"
PN7	Self-treatment with hosiery (if needed), skincare, SLD, home-grown exercise programme, education re self treatment. As initial treatment, patient offered written education leaflets ± sleeve, and a lymphoedema-specific exercise class (small fee).	Alter hosiery prescription to reduce oedema where possible. Offer SLD clinic to help those struggling to do SLD. Intensive-DLT includes compression bandaging, skin care, MLD and exercise; colleague is not trained to provide MLD, so given IPCT and kinesiotaping instead of MLD. Approx. 6 week wait.	Ensuring good fit of hosiery and ability to apply garments is necessary for adherence. Getting them to exercise regularly may also help adherence to prescribed hosiery.	Will book DLT if think there is clinical need (criteria n.d.) *"hope that it would continue to improve, without the patient having to come in and attend for intensive treatment."*	Decided to refer to 'self-treatment phase', because intentionally uses heavier hosiery to reduce swelling. "*I prefer the term self-treatment phase, because I use sleeves as treatment on some patients. (…) I might give someone a sleeve that is quite heavy duty for them to help reduce, and then have them back and we'll re-measure and get a smaller one. But because they're not in CDT phase it's not termed as treatment. But it is; it's self-treatment.*"

Theme 1: (practitioner's individual responses): Usual care offered to women with BCRL (FGP3)

Case ID	1.1 'Usual' lymphoedema treatment	1.2 Variations to 'usual' BCRL treatment	1.3 Women's adherence to recommended treatment	1.4 When is intensive-DLT offered?	1.5 What does the term 'treatment' mean?
PN8	Self-management with hosiery, exercise, skincare ±SLD. Prefers to teach exercise than SLD as exercise has wide range of benefits for wellbeing & lymphoedema.<br>Mostly uses garments available on prescription.<br>Initial treatment is: Self management with hosiery, exercise, skincare ±SLD.	Adjust hosiery prescription; may start with hosiery they can tolerate rather than what is clinically best. May add ACWD.<br>Intensive-DLT comprises 2x/wk Coban2 bandaging + MLD. May add kinesio-taping and occasionally IPCT.	Consider what treatment they can apply & tolerate & adjust recommendations accordingly event if not the 'best' treatment.<br>Involve patient in setting goals, especially if have mobility problems. Need to pace instruction and not overloading the patient.	Main criteria for intensive-DLT is EAV>20%	Uses terms 'self management' or 'supported self-care' as working with the patient to initiate how they will do their care.<br>Hope to achieve a reduction (treatment) with hosiery
PN9	Routine treatment at previous job included skincare, exercise, and compression hosiery. Current job also includes SLD and education.<br>Initial treatment is self-management with hosiery, exercise, skincare ±SLD.<br>Expect to get oedema reduction with hosiery.	Adjust hosiery prescription. May add ACWD or substitute ACWD for intensive-DLT. Patients invited to LeBed exercise class which operates like a support group.	Will assess what they are willing to do as better to wear some hosiery than nothing.<br>Patients often have issues other than BCRL and cancer, so use exercise to help with motivation and encourage normal use of arm.	Criteria for intensive-DLT at previous job required EAV≥ 20% and used wait list, whereas in current job is able to offer bandaging and MLD at earlier stage	*"I think we all hope that wearing a sleeve is going to reduce the volume and improve the texture. But I'm not sure if I think of it as a treatment in that way."*

[1]*For participant characteristics, see Table 3b*

Theme 2 (practitioner's individual responses): Monitoring outcomes (FGP1)				
**Case ID**	**2.1 Assessment methods used by practitioners**	**2.2 Challenges for assessment & monitoring**	**2.3 Expectations of treatment**	**2.4 Priority outcomes**
PN1	Uses Perometer to measure arm volume	It is difficult for women to answer questions about impact of swelling at their 1st lymphoedema assessment. *"I think that being confronted with a question like this is not easy in your first initial assessment. But if you take something home, and you're given the time to think about it, and just fill it in as the time goes by, it would give us more information actually."*	2.3.1. [n.d.]<br>2.3.2. Intensive-DLT: will improve function, appearance, QOL. Lack of objective improvement is not necessarily a problem if swelling stable and patient happy.<br>However, if can't reduce swelling then can keep it stable and avoid cellulitis.	Reduced size is key response to treatment. Also important are tissue consistency changes, patient perspective of BCRL impact and quality of life, episodes of cellulitis.<br>A good intensive-DLT outcome is patient satisfaction regardless of physical changes.
PN2	[n.d.]	[n.d.]	2.3.1. [n.d.]<br>2.3.2. Intensive-DLT: Improved QOL, patient happy with range of movement and activities they can resume after treatment. Should aim for stability if cannot gain reduction. *"Overall, I'd be looking at how happy the actual patient is with their day-to-day life after treatment."*	QOL<br>[didn't disagree with suggestions from other participants]

Theme 2 (cont): Monitoring outcomes (FGP1)				
**Case ID**	**2.1 Assessment methods used by practitioners**	**2.2 Challenges for assessment & monitoring**	**2.3 Expectations of treatment**	**2.4 Priority outcomes**
PN3	Monitor fibrosis and mobility. [showed limited knowledge about reliability of different volume measurement methods.]	Many different ways to measure arm volume. No pre-op measurements taken so don't have measures at start of lymphoedema journey.<br>*"Sometimes you sit there and think, 'Is this swelling there?' but the patient is saying how uncomfortable it is, so you're trying to work out: Is there something else going on here? Is this more to do with the nerves or with the mobility of shoulder?"*	2.3.1. [n.d.]<br>2.3.2. Intensive-DLT: Aim for stability, to improve arm shape to better cope with hosiery, and able to self-care. Doesn't want to give false hope that arm will return to normal or swelling will resolve with treatment. A good DLT outcome would be to reduce the arm to minimal swelling or >10% reduction and maintain improvement at least 3-6 months post DLT.	Arm volume, also tissue tone (fibrosis), patient perspective of swelling severity, and monitor mobility/ROM.<br>Could be helpful if patient completes a diary of how they feel their arm doing.
PN4	Asks patients to complete diary of perceived treatment progress. Mentioned tonometry and had heard of moisture meter & ultrasound. [displayed limited awareness of range of available assessment tools]	[n.d.]	2.3.1. Self-treatment: Outcomes very individual. Expect some improvement within 3 months of wearing hosiery, and a good reduction with soft oedema. If very firm, fibrosed oedema, may soften tissues & improve QOL but unlikely to reduce size.<br>2.3.2. Intensive-DLT: reduce >5% EAV, soften fibrosis, improve QOL. Really disappointing when little/no effect, so would check for other pathology. Don't want to give false hope re outcomes.<br>*"Sometimes you have a soft oedema that reduces really easily and then a good result would be >5% reduction, or hopefully something as significant as 10%. But for some patients who had a very firm oedema and fibrous tissue, sometimes your reduction is not much but the texture of the tissue has softened and it improves the QOL for the patient and that is a good result."*	Agreed with colleagues re arm volume, tissue tone (fibrosis), patient perspective and using a patient diary of feelings about arm and impact of stress levels.

Theme 2: Monitoring outcomes (FGP2)				
**Case ID**	**2.1 Assessment methods used by practitioners**	**2.2 Challenges for assessment & monitoring**	**2.3 Expectations of treatment**	**2.4 Priority outcomes**
PP1	LYMQOL	Unsure whether patients with early LO can reasonably answer LYMQOL questions, if they know what impact LO has on them.<br>Unsure how to capture benefit when there is no obvious physical improvement but the patient reports feeling better	General goals of Rx not discussed.<br>2.3.1. self-treatment: [n.d.]<br>2.3.2. intensive-DLT: Implied patient may feel significant improvement with intensive-DLT even when little volume reduction. Expects most benefit (reduced volume) in 1st week, and potential rebound swelling post-intensive-DLT.	Impact of BCRL on their lives.<br>Subjective view of change.
PP2	Tissue tone.<br>Has used MYMOP to monitor MLD benefit.	"*We've always struggled with outcome measures."*<br>Hand is difficult to measure.	Goals and expectations are adapted to what patients want.<br>Patients want MLD or surgery.<br>2.3.1. self-treatment: Some patients will reduce swelling with appropriate hosiery. Prevent cellulitis.<br>2.3.2. intensive-DLT: [n.d.]	Bioimpedance, tissue tone, change in individual symptoms, e.g. ache, heaviness, fullness.<br>Long-term retention of intensive-DLT benefit.
PP3	Arm measurement method not reported. Palpate tissues.	[n.d.]<br>[Asked about how to use & interpret BIS]	[n.d.]<br>Patients want/expect to receive MLD.	Monitor fibrosis (using tonometry).
PP4	Arm measurement method not reported. Suggests considering photography and maybe HAD scale to assess outcomes	[n.d.]	2.3.1. self-treatment: Should be possible to decongest limb if doing exercise, SLD and self-bandaging or wearing Velcro wrap over hosiery<br>2.3.2. intensive-DLT: [n.d.]	Number of cellulitis episodes.
PN5	Arm volume (tape measure), tissues, QOL questionnaire, questionnaire to identify needs	[n.d.]	[n.d.]	Arm volume

Theme 2: Monitoring outcomes (FGP3)				
**Case ID**	**2.1 Assessment methods used by practitioners**	**2.2 Challenges for assessment & monitoring**	**2.3 Expectations of treatment**	**2.4 Priority outcomes**
PN6	Arm volume (Perometer) to measure size and shape distortion, pitting, tonometry to monitor fibrosis	Difficulty identifying minimal oedema. Variability of Perometry measurement position. No tool to objectively measure pitting oedema.<br>Bioimpedance changes don't make sense so not sure how useful for monitoring changes.<br>Unsure if MYMOP would be useful in proposed study as patients identify different issues.	2.3.1. Self-treatment: Previously thought goal was maintenance, but now expect early/mild oedema to improve with hosiery alone. *"We've got more options for the garments, different materials, and also our patients are being referred a lot earlier…so there's now more opportunity to make an improvement with compression garments alone" ((PN8 and PN9 nods))*<br>Expect exercises to help form lymph and improve lymph transport as well as maintain mobility.<br>2.3.2. intensive-DLT: should improve fibrosis and skin, normalise arm shape, soften dense tissues; improve comfort and movement.<br>Treatment will not resolve underlying issue of missing or damaged lymphatics. So, aiming to achieve latency, no visible swelling, may not be realistic goal.	Volume, pitting (although lack objective measure), fibrosis, treatment cost. Perhaps distress thermometer to monitor study impact on patients.<br>A good intensive-DLT outcome would be to reduce from mild to latent oedema.
PN7	Used MYMOP in another study [other tools n.d.]	Although MYMOP found a common issue, so useful, the identification of many different issues was difficult to interpret.	2.3.1. Self-treatment: Aim to optimise swelling, reducing size and symptoms with range of hosiery (particularly flat-knit), but at minimum to not worsen. Exercise used to maintain mobility and increase confidence to return to normal, while also opening lymphatic pathways. *"If you're doing maintenance, but hoping to get improvement, it's a more gradual thing."*<br>2.3.2. intensive-DLT: expect a more drastic improvement. *"Normally you're looking for a more drastic improvement (with intensive-DLT) than you would expect to get just with maintenance."*	[n.d.]<br>A clinically significant difference would be controlled swelling with minimal hosiery.

Theme 2: Monitoring outcomes (FGP3)				
**Case ID**	**2.1 Assessment methods used by practitioners**	**2.2 Challenges for assessment & monitoring**	**2.3 Expectations of treatment**	**2.4 Priority outcomes**
PN8	Arm volume.<br>Found distress thermometer useful [other tools n.d.]	There are multiple aspects to treatment so difficult to know whether treatment outcome is due to hosiery, or other component such as exercise.	2.3.1. Self-treatment: Expect reduced oedema with self-care. Prevent cellulitis, reduce size and soften fibrosis, maintain good skin condition and soft subcutaneous tissues, maintain mobility, enable to successful live with BCRL & do self-care.<br>Use exercise to maintain mobility and open up lymphatic pathways.<br>*"I do feel that we do get a reduction in oedema by using compression hosiery…I would expect to get a reduction if they're coming through (referred) so quickly and the oedema is fairly new."*<br>2.3.2. intensive-DLT: [n.d.]	Time to discharge, proportion with resolved symptoms or not needing hosiery to control swelling.<br>A good, clinically significant DLT outcome would be to get rid of oedema and discharge patient with no/minimal hosiery.
PN9	[n.d.]<br>Agreed with others	[n.d.]<br>Agreed with others	2.3.1. Self-treatment: using hosiery will reduce volume and improve texture. Exercise will maintain arm mobility and open up lymphatic pathways.<br>2.3.2. intensive-DLT: to reduce arm to minimal/ no oedema would be a good, clinically significant outcome.	Initially stated 'don't know'. After listening to discussion, suggested patients would want arm to return to comparable size to unaffected arm. "*It's like the aim that they had for the surgery, isn't it? The aim for the micro surgery was that they wouldn't have to be in the heavy arm; that it would reduce it enough for it to be more like their other arm really. That's what they want."*

Theme 3 (practitioner's individual responses): Acceptability of proposed study (FGP1)			
**Case ID**	**3.1 Acceptability of proposed lymphoedema trial**	**3.2 Burden of proposed study interventions**	**3.3 Potential benefit of initial intensive-DLT**
PN1	Supports need for study. Research will raise the profile of lymphoedema management.<br>However, as already have waiting lists for people requiring practitioner-delivered treatment, whether DLT or with bandaging or MLD only, there are resource issues for providing study intervention.<br>Unsure of effectiveness of IPCT compared to intensive-DLT. No experience of using pneumatic pumps, as reviewed literature and decided it wasn't cost-effective.	Need to monitor bandaging pressures to ensure not too tight.<br>Consider timing of treatment as thinks women should first come to terms with BCRL and then consider DLT: '*I think they need to deal with the lymphedema first and being aware of what is going on in their body and how they can manage it*.'	Believes women should be offered DLT as soon as possible to prevent irreversible tissue changes that make treatment very difficult.<br>*"I think that if patients with confirmed lymphedema were offered DLT at the first contact with the clinic it would have a very beneficial effect, because we do see the changes as time goes by… creating the cementing effect within the tissue and this cannot be reversed as … it leads into the hard type of tissue and … there's no way to drain the fluid through that... So the sooner the better I think."*
PN2	Considered patients more likely to participate in study if they received personal invite suggesting they had been selected/ chosen to participate.<br>[displayed lack of knowledge about selection process for trials]<br>Patient may not be psychologically ready for intensive-DLT, and many patients don't want bandaging.<br>Thinks IPCT (study intervention 2) would be more acceptable to patients.	Burden/dislike of compression bandaging. Coming for daily DLT may be reminder of cancer.<br>Concerned women may not wear hosiery between IPCT treatments.	Offer DLT to everyone who needs it, but consider whether woman is ready for it.<br>*"my first reaction would be yes, offer it to everyone, but I wonder if there would be a timeframe within which it can be offered because I think many patients psychologically just aren't ready to take on that level of treatment. Maybe it's better to wait a short while when (sic) they've had more time to adjust and then take it on."*

Theme 3 (cont): Acceptability of proposed study (FGP1)			
**Case ID**	**3.1 Acceptability of proposed lymphoedema trial**	**3.2 Burden of proposed study interventions**	**3.3 Potential benefit of initial intensive-DLT**
PN3	Believes there is a need for research to find out what treatments really work, how to make best use of time and clinic space, and find alternative ways to manage limited resources. But unsure of evidence for intensive-DLT and IPCT [displayed lack of knowledge regarding IPCT]<br>May be easy to introduce trial at first assessment as many women already aware of various clinical studies. Depends on readiness for DLT.<br>Need finances and time for practitioners to participate in study.	Time commitment.<br>Bandaging will cause difficulty with daily activities, e.g. cooking.	As there is a lack of evidence and many women with recent-onset symptoms respond to hosiery, thinks it would be better to first determine whether BCRL or transient oedema, and monitor effect of self-treatment first.
PN4	The trial is a great opportunity to measure the effect of intensive-DLT. Limited experience with IPCT but think there's no evidence for using it and likely to be less effective than intensive-DLT with compression bandaging.<br>Agreed that women should be given study information and opportunity to decide whether to participate in lymphoedema research regardless of practitioner opinion of woman's readiness to participate. [changed opinion]<br>*"My initial reaction was no, no, no! But then, in truth, they can just reject it if they don't feel like it. So it gives them the option that they could opt in or opt out."*	[n.d.]	Good idea to offer early DLT. Thinks women would be keen to have early DLT and would cope with early DLT if in right frame of mind.<br>Will reduce risk of tissue fibrosis and hardening of tissues.

Theme 3 (cont): Acceptability of proposed study (FGP2)			
**Case ID**	**3.1 Acceptability of proposed lymphoedema trial**	**3.2 Burden of proposed study interventions**	**3.3 Potential benefit of initial intensive-DLT**
PP1	Likely to be able to recruit to trial, especially with modified DLT which they think will be more acceptable to women.<br>Services lack capacity to treat patients who need DLT, so no capacity to deliver the study intervention. Practitioners may be unwilling to offer DLT to trial patients who don't fit their clinical criteria.<br>*"You might find clinicians who feel they're not able to provide the intensive treatment to those they want to, so to then be offering it to those that would not meet their criteria!"*	Daily travel and time commitment of extra appointments.	Would like to know if early DLT is best treatment. However, thinks it might be better to wait and see who needs DLT as many patient's symptoms remain mild with just hosiery and self-treatment, and patients don't mind sleeve because it doesn't interfere with life.<br>Patients want maximum treatment (eg MLD) due to fear of worsening BCRL. Giving early intensive-DLT could raise expectations of further courses of treatment.<br>Agreed with PT3 that DLT may not be appropriate or acceptable for all patients.
PP2	Think patients would be interested in taking part in study as already interested in other treatments.<br>They would have a right to decline study participation.<br>*"I think you could (recruit) for the modified (intervention)."*<br>Unsure about capacity to deliver intervention as clinic involvement in research may require a reduction of clinic caseload.<br>Could ask questions whether service offers optimal treatment and expectations that should receive intensive-DLT	Main problem would be coming to appointments.	Early DLT may keep oedema at minimal severity and avoid developing typical complications.<br>Unless symptoms are severe, self-care with adherence to treatment should be considered before offering intensive-DLT
PP3	Did not discuss acceptability of study interventions.<br>Concerned about limited resources and site capacity to deliver study intervention	Patients don't want bandaging: as it impacts on work, home life, children.	Believe some pts just want a sleeve and to carry on with life so wouldn't find early intensive-DLT acceptable. They may not be psychologically ready for intensive-DLT and its impact on daily life.

Theme 3 (cont): Acceptability of proposed study (FGP2)			
**Case ID**	**3.1 Acceptability of proposed lymphoedema trial**	**3.2 Burden of proposed study interventions**	**3.3 Potential benefit of initial intensive-DLT**
PP4	Unsure whether patients would find bandaging acceptable or need time to get first used to idea of intensive-DLT.<br>Views the proposed study as a comparison of 'treatment' with 'management'. Unsure of interactive effect of treatment components, e.g. MLD, IPCT, hosiery.<br>Challenge with providing intervention as services lack resources to offer intensive-DLT.	Mainly concerned with impact of compression bandaging on daily life and ability to work. Think risk of attrition due to women not completing the bandaging period.<br>Suggested using ACWD instead of bandaging as it would allow women to continue work during intervention period.	DLT would reduce/prevent fibrosis and thickening of tissues. Important to provide early DLT as a delay, with self-care, may lead to fibrotic or thicker tissues needing DLT to soften them.<br>*"I do believe the important thing is early treatment, because if you just put them in the sleeve and then you can't see them for another few months they could have developed fibrosis by then or deep thickening of the tissues, and then it becomes a battle because you've got no spaces and you can't book them in for intensive treatment."*<br>Thinks newly diagnosed patients should be offered more group education about lymphoedema and its treatment. "*Maybe we should be running education classes for people when they're first diagnosed"*
PN5	Think most patients likely to be willing to participate, particularly as they may already be used to clinical trials.<br>Acceptability of proposed interventions n.d.<br>Trial could highlight differences in service provision in NHS and private health care facilities.	[n.d.]	Thinks experts are suggesting early-intensive-DLT is beneficial but evidence is lacking.<br>*"Is that evidence going to come in and give us the fact that actually you do need to decongest them at a very, very early stage? I think the evidence is slowly coming, but at the moment we haven't got it."*

Theme 3 (practitioner): Acceptability of study (FGP3)			
**Case ID**	**3.1 Acceptability of proposed lymphoedema trial**	**3.2 Burden of proposed study interventions**	**3.3 Potential benefit of initial intensive-DLT**
PN6	Thinks women may decline if not ready for time commitment, particularly if recently completed cancer treatment.<br>Clinic has <25 BCRL referrals per year, so wouldn't be able to recruit many patients at that site.<br>Acceptability of intervention n.d. Does not currently use IPCT.<br>Suggests 3-month self-treatment prior to intensive-DLT to identify those not needing it, as has seen improvement within 3-month but not 1-month.	Time commitment as might not want to come every day. Timing may be wrong for anyone completing cancer treatment.	Questioned whether early intensive-DLT is necessary as sees progress with other modalities, e.g. hosiery, exercise and movement.<br>Early DLT won't address the underlying problem of disrupted lymph pathways.<br>*"None of this treatment is going to do anything to resolve the whole reason why they've got lymphoedema in the first place … So, whether we give them ten treatments at the beginning or ten treatments over 24 months we've still got the problem of a long-term and chronic issue because we've disturbed lymph function.*
PN7	Thinks trial might be acceptable if rationale for early DLT is clearly explained. Women unlikely to be keen to have bandaging if only have minimal oedema.<br><br>Clinics lack capacity for practitioners to provide study treatments as currently unable to provide daily DLT and have wait list.	Time commitment - difficult to commit to 5 days per week.<br>Travel.<br>Women may be unhappy if have minimal oedema and are allocated to bandaging.	Early DLT could prevent development of lymphoedema-specific changes and associated difficulty with treatment outcomes.<br>*"I think there are a lot of specialists out there that feel the same: that would like to get in earlier and try and decongest earlier before it starts building up and causing the changes that we then have to struggle to manage."*

Theme 3 (practitioner): Acceptability of study (FGP3)			
**Case ID**	**3.1 Acceptability of proposed lymphoedema trial**	**3.2 Burden of proposed study interventions**	**3.3 Potential benefit of initial intensive-DLT**
PN8	Questioned whether one month is long enough to gauge progress with self-treatment.<br>Some women may find it too soon after cancer treatment to consider intensive-DLT.<br>Services may use different bandage systems, e.g. Coban<br>Trial could raise patient expectation of receiving practitioner-delivered treatment as routine care.	Patient may want to get back to work.	Questioned whether early intensive-DLT would achieve better results than hosiery alone, and if worth treating minimal oedema as hosiery would be required to maintain any treatment benefit.<br>*"I suppose what we're saying is we've got patients that we're discharging say at two years, but they've actually been in quite lightweight hosiery all along because their oedema has been very minimal. So, if we're going to put them through all of that then you'd want something less than what we've got already."*
PN9	Acceptability of proposed study interventions n.d.<br>Lack of service capacity to deliver study intervention. Would increase delay obtaining DLT.<br>*"I always felt I was doing things back to front a little bit, not being able to give the MLD and the bandaging at the beginning. But I've got no research or anything to back that up; it's just how it felt, that's what I wanted to do." ((group agreement))*	[n.d]	Will get best result with DLT given at earliest point.<br>*"you know you'd get a better result if you got in there a little bit earlier. Then the sleeve is smaller."*<br>"*I always felt I was doing things back to front a little bit, not being able to give the MLD and the bandaging at the beginning."*

Theme 4 (practitioner's individual responses): Lymphoedema service issues (FGP1)				
**Case ID**	**4.1 Characteristics of women newly presenting for BCRL treatment**	**4.2 Changing nature of lymphoedema practice**	**4.3 Challenges and frustrations of managing limited resources**	**4.4 Patient education**
PN1	Some have visible, palpable swelling, others do not.	[n.d.]	Have separate waiting lists for patients who need DLT or just MLD or bandaging.	Important to invest time in teaching patient to understand lymphoedema and how to manage it, to engage patient and improve concordance. Amount of information can be overwhelming.<br>Think there is lack of info for patients re chronic nature of lymphoedema.
PN2	[n.d.]	[n.d.]	[n.d.]	[n.d.]
PN3	Seeing more hand and finger swelling<br>Need to determine if oedema transient or BCRL.	Can be difficult to identify cause of complex lymphoedema.	Need to find better ways to manage limited resources and reduce treatment costs.	Patients need information re treatments.<br>Patient education provided over time, as too much information for one visit. Prefer gentle approach as can overload patients by giving too much info at one session. So will teach SLD at subsequent visit.
PN4	Some may present with firm oedema from start, e.g. hard tissues with no soft oedema	Changing BCRL characteristics n.d.	Using Coban system twice weekly rather than daily bandaging to manage lack of clinic capacity.<br>No budget for hosiery so patient must use GP prescription/ pharmacy.	Some people want lots of information (implying that others don't).<br>Lack of patient knowledge will affect understanding of treatment. Patients mistakenly think MLD will fix their swelling.

Theme 4 (cont): Lymphoedema service issues (FGP2)				
**Case ID**	**4.1 Characteristics of women newly presenting for BCRL treatment**	**4.2 Changing nature of lymphoedema practice**	**4.3 Challenges and frustrations of managing limited resources**	**4.4 Patient education**
PP1	Women present earlier with less complicated BCRL.	Sees patients earlier, so lymphoedema not so complicated.<br>Hosiery largely obtained via GP prescription to reduce cost and facilitate discharge.	Happy with treatment for mild oedema but lack resources to offer intensive-DLT to those with moderate-severe BCRL who need it.<br>Pressured to obtain hosiery via GP prescription/local pharmacy to reduce clinic costs, so unlikely to fit patient in non-FP10 garment unless nothing else suitable.<br>*"We're increasingly under pressure for as much as we can get on prescription. So, if a garment is not available on prescription we're unlikely to start putting a patient in it, unless there really is no other alternative."*	Education to address lymphoedema, nature of chronic problems, and self-management. Often the patient doesn't understand the chronic nature of LO.
PP2	[n.d]	[n.d.]	Have to ration treatment, e.g. MLD, DLT limited to those with severe symptoms (>30% arm volume).<br>May soon need to implement discharge policy to manage growing caseload.	Uses 'Physio Tools', Macmillan & LSN leaflets to support patient education about understanding symptoms and actions to take.<br>Teaches SLD.
PP3	Present quite early. Lately they have really mild oedema.	[n.d.]	[n.d.]	Teach about lymphoedema and self-management. Teach SLD.<br>*"We are really, really into explaining that it's very important to self-manage, and that they understand that we are here to support them but they actually need to do their part. So, we are focusing on that they do really understand what is everything about."*

Theme 4 (cont): Lymphoedema service issues (FGP2)				
**Case ID**	**4.1 Characteristics of women newly presenting for BCRL treatment**	**4.2 Changing nature of lymphoedema practice**	**4.3 Challenges and frustrations of managing limited resources**	**4.4 Patient education**
PP4	[n.d.]	[n.d.]	Unable to routinely provide early intensive-DLT and so patient can develop fibrosis. *"you've got no spaces and can't book them in for intensive treatment."* Can only provide one garment and other has to be bought or obtained via GP prescription.	Uses LSN leaflets and own handouts to educate patient about their responsibility for managing BCRL. Patients need group education classes.
PN5	[n.d.]	More services need to do pre-surgery measurements	Hosiery provision limited by need to use hosiery on drug tariff. No nurse prescribers in clinic so have to request hosiery via GP.	Sends out leaflets (own and LSN) prior to 1st appointment so patients come with some awareness of lymphoedema. Provides workshop with education about self-management, exercise, SLD, skin care, weight management, diet, plus includes 30 min Tripudio exercise class.

Theme 4 (cont): Lymphoedema service issues (FGP3)				
**Case ID**	**4.1 Characteristics of women newly presenting for BCRL treatment**	**4.2 Changing nature of lymphoedema practice**	**4.3 Challenges and frustrations of managing limited resources**	**4.4 Patient education**
PN6	Usually soft oedema, no palpable fibrosis. Shape usually very good, very little skin changes. Volume can vary greatly, e.g. >5%EAV, sometimes hardly any detectable swelling but patient aware of swelling.<br>Agrees with PN7 that many present with breast oedema.	Now present with mild symptoms, many with minimal swelling.<br>Have more hosiery options.<br>Used to do much more intensive treatment because more people presented with severe swelling.<br>*"Our patients are being referred a lot earlier. I'm not seeing the severities, and it's much easier to treat oedema that has developed within the last three or six months than it is within the last 3-6 years." ((general agreement))*	[n.d. Separately mentioned that no restrictions to hosiery use and no requirement to discharge patients.]	Clinic developed own exercise programme and leaflet.<br>Also access to local Macmillan information centre.
PN7	Women mostly presenting with breast+arm swelling. Usually have 10-15% EAV and some breast oedema, or present with significant breast oedema plus mild arm swelling. Breast oedema often not referred as considered DXT reaction despite being present for >6m, with referral prompted by hand or wrist swelling.	10 years ago, the majority presented with arm swelling, few with breast oedema. Now many more patients present with breast oedema as main or only symptom.<br>*"I see a predominance of breast these days; whereas ten years ago it was arms. (…) I'm seeing so much more breast, or (women with) very minimal arm but quite significant breast oedema."*	Would like to offer intensive-DLT earlier, but depends on workload. Has limited capacity as colleague not trained in MLD so cannot offer full intensive-DLT. Prioritises urgent cases with dedicated treatment slots.<br>Has to discharge patients at 18-24 months to manage workload, but may return if GP can't manage hosiery.<br>*"We had a discharge policy foisted on us because we were at breaking point... Of course that does lead to problems with people bouncing back if the GP is unable to manage it. (…) We actually have it on our discharge sheet now that this is what we're recommending. If you (GP) choose to provide an alternative on prescription, then we can't be held responsible for what could happen"*	Use range of Macmillan and LSN leaflets to create personalised education pack.<br>Offer SLD training as 1-1 session to include carer.

Theme 4 (cont): Lymphoedema service issues (FGP3)				
**Case ID**	**4.1 Characteristics of women newly presenting for BCRL treatment**	**4.2 Changing nature of lymphoedema practice**	**4.3 Challenges and frustrations of managing limited resources**	**4.4 Patient education**
PN8	Most have <10%EAV, good tissues, soft, no sign of any fibrosis. The elderly with late presentation of recurrent disease often have significant swelling.	Used to treat many more patients with DLT, but now they are referred more quickly and often with minimal oedema (implies patients used to present with more severe symptoms).<br>*"When I first came into post we did far more DLT than our service does now. I think that's all to do with patients being referred through so quickly to the service, and us being able to see them reasonably quickly. I do feel that we get a reduction in oedema by using compression hosiery."*	Coban system used to treat twice weekly as lack resource for daily DLT<br>Use hosiery available on prescription to reduce cost to clinic. Have nurse prescribers.<br>Discharge patients at 18-24 months if <10% volume and stable, with access back if problems<br>*"It's the only way we would be able to cope with being able to give our time to the new patients coming through, because we just wouldn't have the capacity to keep everybody on."*	Use Macmillan and BCC leaflets as regularly updated, also PhysioTools for exercise.<br>Many patients come with information from BCN, so aim to add to existing knowledge.
PN9	Previous job saw minimal oedema. Current job see mostly soft oedema, <10%EAV. Tends to be older group who are less aware of swelling although feel rather than see something. Late presenters have worse symptoms.		Cost considerations when deciding hosiery.<br>Frustrated that can't offer early intensive-DLT to prevent problems and get best result.<br>*"I did feel that it should be intensive treatment no matter what. (…) That's the first bit you're doing and you've got the best result you can at the earliest point. And then if you can only offer garments after that in your service that's what you have to do. But I always felt I was doing things back to front a little bit, not being able to give the MLD and the bandaging at the beginning." ((group agreement))*	Home-developed general leaflet, SLD, exercise. Also use LSN and Macmillan leaflets.<br>LeBed exercise class.<br>Support group.

Title: Women's and practitioners' views of early intensive decongestive lymphoedema treatment for breast cancer-related arm lymphoedema: a focus group study.

Journal of Cancer Survivorship.

Authors: Jeffs E, Ream E, Chang YS, Taylor C, Bick D. corresponding author: Jeffs E. King's College London and Guy's & St Thomas' NHS Foundation Trust.
Eunice.jeffs@kcl.ac.uk; Tel: 07557325642
