# Supplementary material for: Women’s and practitioners’ views of early intensive decongestive lymphoedema treatment for breast cancer-related arm lymphoedema: a focus group study
Source: J Cancer Surviv. 2025 Dec 2;20(5):2460–73. doi: 10.1007/s11764-025-01897-z (PMC13723537; doi:10.1007/s11764-025-01897-z)
Supplement: Supplementary file 6 [file 11764_2025_1897_MOESM6_ESM.pdf]

Supplementary Information 6: Characteristics of participants at validation event

		Women (n=7)	Practitioners (n=4)
Women's characteristics			
Age group:	• 40-59 years<br>• 60-75 years	2<br>5	
Employment:	• Part-time, full-time work<br>• Retired, not working	1<br>6	
Swelling:	• Unilateral arm<br>• Breast oedema	7<br>1	
BCRL duration (years), *Md* (range)		7.0 (0.5-12.0)	
Perceived severity:	• No swelling<br>• Mild swelling<br>• Moderate swelling	2<br>3<br>2	
BCRL treatment received:	• Self-care only<br>• DLT, IPCT, MLD (+self-care)	5<br>2	
Ethnicity:	• White British	7	
Practitioner's characteristics			
Profession:	• Nurse<br>• Physiotherapist		3<br>1
Lymphoedema service:	• Any Qualified Provider<br>• Community-based<br>• Hospice+community-based<br>• Hospital-based		1<br>1<br>1<br>1
Patients treated:	• Cancer only<br>• Cancer, non-cancer		3<br>1
Experience treating BCRL >5 years			4

Title: Women's and practitioners' views of early intensive decongestive lymphoedema treatment for breast cancer-related arm lymphoedema: a focus group study.
Journal of Cancer Survivorship.

Authors: Jeffs E, Ream E, Chang YS, Taylor C, Bick D. corresponding author: Jeffs E. King's College London and Guy's & St Thomas' NHS Foundation Trust. Eunice.jeffs@kcl.ac.uk; Tel: 07557325642
