# Supplementary material for: Women’s and practitioners’ views of early intensive decongestive lymphoedema treatment for breast cancer-related arm lymphoedema: a focus group study
Source: J Cancer Surviv. 2025 Dec 2;20(5):2460–73. doi: 10.1007/s11764-025-01897-z (PMC13723537; doi:10.1007/s11764-025-01897-z)
Supplement: Supplementary file 7 [file 11764_2025_1897_MOESM7_ESM.pdf]

Supplementary Information 7: Preliminary findings presented to participants

Theme/subtheme	***Key points***
Treatment provided as 'usual' care	• Self-treatment, focus on hosiery<br>• Women and practitioners report treatment variation mostly relates to hosiery<br>• Hosiery often provided through GP/pharmacy
Intensive treatment is not 'usual' care	• Course of treatment with bandaging and MLD<br>• All practitioners delivered intensive-DLT but rationed to those with greatest need/severity of swelling<br>• Considered optimal treatment by women and practitioners<br>• Few women had received any form of intensive treatment
Information and education	• Provided 1-1 or group session as 'usual' care<br>• Women want more information about lymphoedema, treatment options, how to recognise and respond to changes, long-term implications<br>• Women want *written* information about their treatment and what is happening to them, to better self-treat BCRL
Terminology	• What is treatment?<br>• Variable words used to describe treatment<br>• Checking patients and practitioners mean same thing with same words, e.g. exercise–what type of exercise?
Treatment decisions	• Influenced by resources/capacity<br>• Use of clinical criteria and waiting lists to ration treatment, particularly intensive-DLT<br>• Practitioners frustrated by resource constraints
Challenges of hosiery	• Hospital versus GP/pharmacy provision<br>• Delays and incorrect garments affect swelling control<br>• Practitioners issue disclaimers to GP<br>• Keeping garments clean, having enough items
Measurement/ outcomes	• Monitoring size-tangible<br>• Uncertain whether HRQOL tools are useful<br>• Patients unclear what issues should be monitored by PROMS, especially identifying LO-related issues
Expectations of treatment and goal-setting	• Control versus swelling reduction<br>• What is a good outcome?<br>• What can be achieved with self-treatment?
Discharge	• Different service policies<br>• Patients generally not keen to be discharged; they want ongoing monitoring of symptoms/progress
Offering intensive-DLT at early stage	• Varying views of practitioners: expectations of outcome, capacity, varying hosiery to reduce swelling, psychological aspects<br>• Window of opportunity to affect swelling while mild<br>• Burden of treatment<br>• Is BCRL priority? Focus on getting on with life<br>• Modified DLT may be more acceptable, e.g. ICPT

Title: Women's and practitioners' views of early intensive decongestive lymphoedema treatment for breast cancer-related arm lymphoedema: a focus group study.

Journal of Cancer Survivorship.

Authors: Jeffs E, Ream E, Chang YS, Taylor C, Bick D. corresponding author: Jeffs E. King's College London and Guy's & St Thomas' NHS Foundation Trust. Eunice.jeffs@kcl.ac.uk; Tel: 07557325642
